# Beyond borders: Exploring the challenges of refugee children in Saudi Arabia and Turkey

**DOI:** 10.1371/journal.pone.0334841

**Published:** 2025-11-19

**Authors:** Thoraya A. bin Kadasah, Ghadah S. Alsedrani

**Affiliations:** Early Childhood Department, King Saud University, Riyadh, Saudi Arabia; University of Alabama at Birmingham, UNITED STATES OF AMERICA

## Abstract

The number of displaced individuals worldwide is increasing due to revolutions, wars, and economic crises. However, research on the educational, social, and cultural challenges facing refugee children is limited. Thus, this study aimed to investigate these challenges in Saudi Arabia and Turkey by focusing on refugee children’s adaptation to new cultural and social norms. Using quantitative methods, we surveyed 418 parents of refugee children living in Saudi Arabia and Turkey and examined variables such as gender, residency, and parental education level. The findings indicated that the challenges refugee children faced were significantly associated with parental education levels, but not with country of residence. Specific cultural challenges also emerged, highlighting the struggles faced by refugees worldwide and underlining the crucial need for cultural acceptance and resources to facilitate refugee children’s participation in social and cultural settings and their integration into host societies. These findings carry important implications for policymakers in Turkey, Saudi Arabia, and comparable settings, specifically concerning the need for policy reforms and targeted support to facilitate the integration of refugee children into new educational and cultural contexts. This study contributes significantly to current knowledge in that it examined the educational, social, and cultural challenges facing refugee children within the Saudi Arabian context, which had remained largely unexplored in the existing relevant literature, filling a gap in the extant research. See Graphical Abstract.

## Introduction

Because of violent revolutions, wars, and economic crises, the global count of displaced individuals has reached its highest level since World War II at approximately 42.7 million refugees. These displaced individuals, especially children, often face challenges integrating into their host societies [1]. For children specifically, these challenges often affect their education [2]. This situation has compelled host countries such as Saudi Arabia and Turkey and international organizations such as the United Nations High Commissioner for Refugees (UNHCR) and the United Nations to seek sustainable solutions for refugee children, especially concerning their education [3]. That in order for these countries and organizations to succeed in this, it is important to first understand the challenges that refugee children face.

Some of the key factors known to influence refugee children’s educational integration are their personal constraints and families’ socioeconomic statuses. In particular, financial instability, limited parental support, language barriers, and burdensome domestic responsibilities contribute to their disengagement from formal education [4,5]. Concerning socioeconomic factors, existing studies on Syrian refugee children in Turkey have shown that children from low-income households or families with parents who have low levels of education are more likely to not attend school due to financial constraints. For example, Kırdar et al. [4] and Shuayb et al. [6] found that school enrollment rates among Syrian refugee children lag significantly behind those of their local Turkish peers, with gaps of 32.3% for boys and 24.8% for girls—attributable to underlying social and economic disparities. Previous research [7,8] has suggested that addressing these issues through financial aid and community-based support systems can increase school enrollment and enhance children’s overall well-being. Additionally, Mizza [9] asserted that a gradual integration into long-term educational arrangements—such as stable, lifelong learning pathways—can mitigate these challenges and promote consistent educational engagement.

Another factor that is reported to play a vital role in refugee children’s educational experiences is host-country systems. Refugee communities are significantly influenced by the cultural, social, and educational environments of their host societies [10]. The complexities of a multicultural education, limited access to public education systems, and insufficient language support hinder equal participation in learning opportunities, in turn incumbering successful integration. To overcome these challenges, refugee children must adapt to and become proficient in local cultures (cultural adaptation) and languages (language acquisition) while maintaining their own cultural identities, both of which are formidable processes to undertake without sufficient support [11–13].

Moreover, differences in pedagogy and curricula between the host country and refugees’ countries of origin further complicate integration. For example, the percentage of refugee students enrolled in schools in Saudi Arabia, where refugee students must adapt not only to the cultural environment but to the national curriculum as well, is 5.71% [14]. Besides curriculum and pedagogy, Al-Mansour [15] noted that the host country’s cultural diversity can influence children’s identities, sometimes generating friction between traditional values and newly introduced influences, such as foreign media and video games. These cultural tensions raise important questions about tolerance, identity, and acceptance—both within refugee families and host communities.

Prior research has emphasized that enrolling refugee children into host-country education systems without addressing the broader social, cultural, and policy-related challenges can lead to fragmented integration [16,17]. Therefore, it is essential for host nations to adopt inclusive, rights-based approaches that balance respect for diversity with efforts to preserve social cohesion. This involves not only aligning national policies with principles of democracy and human rights but also educating host populations to promote cultural understanding and acceptance [18].

## Scope and study background

Saudi Arabia and Turkey are among the Middle Eastern countries that welcomed numerous refugees. Saudi Arabia alone has welcomed refugees of various nationalities, including Syrians, Yemenis, and Rohingyas, and has offered them schooling, healthcare, and university education opportunities [19]. Saudi Arabia and Turkey also host refugees from other nationalities, amounting to 1.35 million and 3.6 million, respectively [7,20]. However, these host countries grappled with effectively integrating the migrants into society to ensure societal stability and growth. Thus, to overcome some of these challenges, particularly those related to refugee children’s educational requirements, they implemented educational policies to ensure that children have opportunities of both formal and non-formal education at schools [4,7].

By 2023, Saudi Arabia had spent more than 18.6 billion US dollars on refugee integration into society, allowing around 449,000 Syrian refugees living in Saudi Arabia to enjoy career and job opportunities, education, and free healthcare [21]. Of this amount, 7.5 billion US dollars was spent on services provided by the Directorate General of Passports, such as residency, employment procedures, and contracts; 6 billion on healthcare for refugees; and over 5.5 billion on their education [21]. All residents in Saudi Arabia, including migrants, have equal rights to access education, healthcare, and digital government services [22]. Substantial attempts are being made to integrate migrants and refugees into public education schools, reflecting the Ministry’s emphasis on inclusive education [23]. Despite the significant number of refugees in Saudi Arabia (5.5% of the population) and Turkey (3.5 million Syrian refugees) [3], few studies have examined the challenges they face in education.

With both Saudi Arabia and Turkey sharing a contiguous border with Syria, the two countries host about 700,000 [24] and 100,000 [21] school-aged Syrian refugee children, respectively. While both these countries provide the children access to formal education for Syrians, their situations differ. For example, in Turkey, educational services are available to some refugee children at the compulsory school age, which is six years old, leaving many without access to schooling [25], while in Saudi Arabia, all school-aged refugees are generally able to attend school [3]. Acar-Ciftci [16] analyzed Turkey’s evolving approach to integrating Syrian refugee children into its education system. Initially served through Temporary Education Centers (TECs) using Syrian staff and curricula, refugee students began transitioning into public schools starting in 2016. This shift involved policy reforms, language support, legal oversight of TECs, and international collaborations (e.g., through UNESCO, EU). Programs like “Promoting Integration of Syrian Kids” and “Conditional Cash Transfers for Education” supported this effort [16]. The study stresses the importance of evaluating educational approaches through the lens of cultural diversity.

Modern societies are structured around plural communities, where diversity spans social, cultural, and religious backgrounds [3,17]. This diversity gives rise to subcultures that coexist with the broader national culture. Within these communities, native populations interact with groups of diverse origins, contributing to collective community-building. However, refugee communities may face challenges due to displacement from their homelands. Further, countries differ not only in the extent of cultural diversity but also in their approaches to managing it—some embrace diversity through integration and recognition, while others adopt policies that overlook it [11].

There are many other Middle Eastern countries that have received Syrian refugees. For example, in Jordan, there were 673,193 registered Syrian refugees, of whom 126,115 were residing in camps while the number of Syrian refugees in Egypt has reached 133,862 [3]. while simultaneously experiencing various forms of exclusion within host communities. Drawing on the concept of “right to have rights,” refugee children in Middle Eastern countries encounter multiple challenges that hinder their full integration [27]. Nevertheless, many strive to engage with society through what are termed “acts of citizenship,” including pursuing education, acquiring professional skills, establishing organizations, participating in the labor market, and building social networks. Although Syrian refugees in some Arab Regions remain legally excluded from acquiring citizenship, several studies [26,27] demonstrate their ability to negotiate forms of “de facto citizenship,” enabling them to move beyond their transitional condition and open new prospects for their future in exile [26,27].

This situation has led to several educational, cultural, and societal challenges. The curricula and educational approaches adopted for refugee children represent the crux of their educational and pedagogical challenges. When a child’s culture diverges from their homeland, it can affect their identity. Meanwhile, their educational culture tends to align with the curriculum implemented in their host country. This was confirmed by Gezer [28], who highlighted that Turkey has played a major role as the host country for a significant number of Syrian refugees since the outbreak of the Syrian civil war in 2011.

In addition to these challenges, various issues related to rights, democracy, citizenship, and education have emerged [29]. St. Nicholas [30] highlighted the importance of promoting cultural diversity among children in classrooms, stating that working on cultural diversity with children is an adventurous journey that involves sparking curiosity. Consequently, aspects of multiple cultures can be introduced to them by providing knowledge about the culturally relevant aspects of other individuals within the school environment. This includes presenting the religious, artistic, culinary, fashion-related, and even technological aspects of other cultures. Hence, the pedagogical approaches used in childhood education can enrich children’s daily lives by enhancing their knowledge of various practices associated with cultural diversity. Thus, Khalawi and Bidwi [31] recommended developing curricula that embrace the principles and concepts of multicultural education, incorporate cultural diversity concepts, and reinforce their associated values. Additionally, various approaches have been suggested to aid children in understanding the characteristics of other cultures, allowing them to learn by example how to create a healthy environment both at home and in school, thereby enhancing their sense of ethics and respect in their social interactions.

Refugee children and school-age youth face a range of nuanced educational challenges that go beyond access to schooling [2]. These challenges are deeply shaped by their refugee status, including forced displacement and the need to adapt to unfamiliar educational systems. Moreover, these educational challenges emerge when families relocate, a theme explored in several studies. However, the Turkish government is continuing efforts to ensure that registered refugees have access to essential rights and public services, including education and healthcare [32,33].

Through a study involving principals of government schools with refugee students, Caliskan [13] examined the marginalization experienced by refugee students in Turkish schools, given the influx of refugees into the country in recent years. The study results indicated that school principals worked tirelessly to provide refugee students with a socially fair school environment. School administrators have also attempted to ensure social justice [13]. On the other hand, teachers and principals in such settings have reported overwhelmed classrooms, insufficient teacher training, and inadequate curriculum planning, which together hinder refugee student integration and wellbeing [34].

Additionally, communication and language barriers between refugee children and Turkish primary school teachers have been identified as major challenges to educational integration [2]. A qualitative study involving Syrian refugee students in Turkey highlighted the positive impact of peer and teacher support on student motivation. A student shared that when she struggled to understand the material, her Turkish peers and teachers were very helpful, which encouraged her to persevere [25]. In contrast, the same study documented another student who faced the Turkish curriculum without any Arabic language support, leaving her frustrated and demotivated [25].

Beyond language, refugee children often experience social disconnection and cultural shock due to abrupt exposure to unfamiliar norms, further increasing their vulnerability in school settings [35]. Addressing these challenges requires host countries to implement more effective language instruction, employ bilingual or refugee-background teachers, and provide psychosocial support strategies that empower refugee children [36]. Ultimately, refugee acculturation and adaptation are shaped not only by school-level responses but also by the broader social context of the host country [37].

Despite the importance of addressing the cultural, educational, and linguistic challenges refugees and their children face in diverse contexts, as highlighted above, and despite both Saudi Arabia and Turkey emphasizing equal opportunities for refugees, research on the acculturation stress experienced by refugee children—particularly in Saudi Arabia—remains limited [4,38]. Therefore, the authors conducted the present study as a comprehensive descriptive analysis comparing the perspectives of refugee children in Saudi Arabia and Turkey to better understand how refugee integration works. Specifically, the study aimed to explore the educational, social, and cultural challenges faced by refugee children aged 4–6 years to contribute evidence on refugee education and social and cultural welfare in Saudi Arabia. The present study focused specifically on this age because, early childhood particularly the period from four to six years of age—represents a critical stage in the development of personal identity. Thus, targeted early interventions during these formative years can play a pivotal role in fostering a strong sense of belonging and facilitating a smoother, more rapid adaptation to newly encountered communities following migration.

The current research employs the sociocultural theory perspective, formulated by Vygotsky [39], a Russian psychologist, which asserts that cognitive growth is fundamentally influenced by social and cultural environments. Vygotsky stressed that learning is socially facilitated; that is, individuals develop understanding through interactions with those possessing more knowledge—like educators, caregivers, or peers—within their cultural surroundings. For example, early childhood teachers with strong cultural and linguistic skills are better able to support international students, helping to ease cultural shock and improve their learning outcomes.

This theory is centered on the zone of proximal development (ZPD), which refers to the scope of tasks that a child can accomplish with assistance but not independently [40]. In this zone, learning takes place effectively when suitable support, or “scaffolding,” is provided. Considering this, then, interactions between migrant children and their teachers and classmates in the host country can be considered crucial for aiding adaptation, education, and the growth of new cognitive abilities. Collaborative dialogue and culturally responsive teaching practices facilitate children’s co-construction of knowledge, enhance their confidence, and assist them in overcoming the difficulties associated with adjusting to a new educational setting [41]. Thus, applying Vygotsky’s framework could help highlight the importance of supportive, culturally aware teaching practices in promoting the academic and emotional development of refugee and migrant children in Turkey and Saudi Arabia.

However, although both Turkey and Saudi Arabia are Muslim-majority nations with cultural and religious ties to Syria, Turkey is more religiously and ethnically diverse than Saudi Arabia, which follows Islamic principles based on the Quran and Sunnah [42]. Their religious observances also differ, as noted by one of the researchers during a four-year stay in Turkey [43]. Moreover, they exhibit distinct differences in language, social norms, and integration models. The disparity between Turkey’s language barrier and sociopolitical dynamics, on one hand, and Saudi Arabia’s Arabic-speaking environment and more conservative societal structure, on the other, provides a unique opportunity to examine how diverse sociocultural environments influence the integration and adaptation of refugee children.

Thus, this study also sought to determine whether there are statistically significant differences in the challenges faced by refugee children (aged 4–6 years) in Saudi Arabia and Turkey from the parents’ perspective, considering the variables of the country of residence, economic and parental education levels.

This study attempted to answer the following research questions:

(1) What are the educational challenges faced by refugee children residing in Saudi Arabia and Turkey?
(2) What social challenges do refugee children residing in Saudi Arabia and Turkey encounter?
(3) What cultural challenges do refugee children in Saudi Arabia and Turkey face?
(4) How do the challenges encountered by refugee children vary by their country of residence (Saudi Arabia and Turkey), and are they statistically significant?
(5) How do refugee parents’ perspectives (in both countries) statistically differ in terms of the challenges their children faced based on their education level?

## Method

This study adopted a descriptive approach to elucidate the challenges experienced by refugee children aged 4–6 years living in Saudi Arabia and Turkey from their parents’ perspectives. Ali Khan, et al. [44] revealed that this approach in research focuses on providing a detailed account of a situation or group without altering or influencing it. Furthermore, it is concerned with answering what, who, where, and when questions to provide an understanding of the context, patterns, trends, relationships, and environment of the participants. This helps to make generalizations about the larger population based on the sample studied. Simultaneously, by using a quantitative approach, it provides empirical data on the challenges faced by refugee children in Turkey and Saudi Arabia from their parents’ perspectives.

### Participants

The study population included Syrian parents of refugee children aged 4–6 years living in Saudi Arabia and Turkey, and the total sample size was 418 participants. Parents were chosen to be the respondents because they are their children’s primary caregivers and are responsible for their day-to-day care and welfare, making their awareness of and opinions about their children’s challenges important for this study. The refugee parents were of Arab descent (Syrian) and had a Muslim cultural and religious background. The selection of Saudi Arabia and Turkey as the focus countries was intentional, based on their geographical proximity to Syria and their reception of many Syrian refugees. These large communities provide accessible and diverse populations for study, increasing the reliability and relevance of the research findings. Furthermore, one of the researchers has lived in Turkey for several years, and both researchers are currently residing in Saudi Arabia.

Participants were selected using the purposive sampling technique, a type of non-probability sampling, to ensure the inclusion of diverse parental perspectives across key demographic categories. The selection criteria included: type of parent, refugee residency status, parental education level, and years of residency. Efforts were made to include participants from different educational backgrounds and durations of residency to avoid sampling bias and ensure broader representativeness.

Written consent was obtained through a checkbox in the questionnaire stating: “Check the box if you agree to participate in this survey.” This was accompanied by a confidentiality statement: “We assure you that the data will remain confidential and be used solely for scientific research purposes” (Appendix 1). Data were collected using digital surveys via Google Forms. The survey was distributed through multiple channels between 2 April and 20 May 2024. School administrations in Saudi Arabia and Turkey were contacted via email, and these institutions relayed the study invitation—including a detailed description of the research purpose, the consent form, and the survey link—to parents of Syrian refugee children aged 4–6 years enrolled in public and private kindergartens. Additionally, one of the researchers directly contacted potential participants via email and WhatsApp, leveraging prior relationships and community knowledge as an Arab resident in Turkey and a current resident in Saudi Arabia. Then, by applying the snowball sampling technique, the survey was forwarded to all Syrian refugee parents whose 4–6-year-old children were enrolled in public and private kindergartens in Saudi Arabia and Turkey. The participant demographics are presented in Table 1.

**Table 1:** The demographic distribution of the participants based on the host country and parents’ education level.

Variable		Frequency	Percentage
Residency	Saudi Arabia	219	52.4%
	Turkey	199	47.6%
Educational level of the fathers	Pre-university	130	31.1%
	Undergraduate	201	48.1%
	Postgraduate	87	20.8%
Educational level of the mothers	Pre-university	98	23.4%
	Undergraduate	241	57.7%
	Postgraduate	79	18.9%
Years of residency	Less than 1 year to less than 3 years	46	10.8%
	3–6 years	96	23%
	More than 6 years	276	66%
Total		418	100%

**Fig 1:**
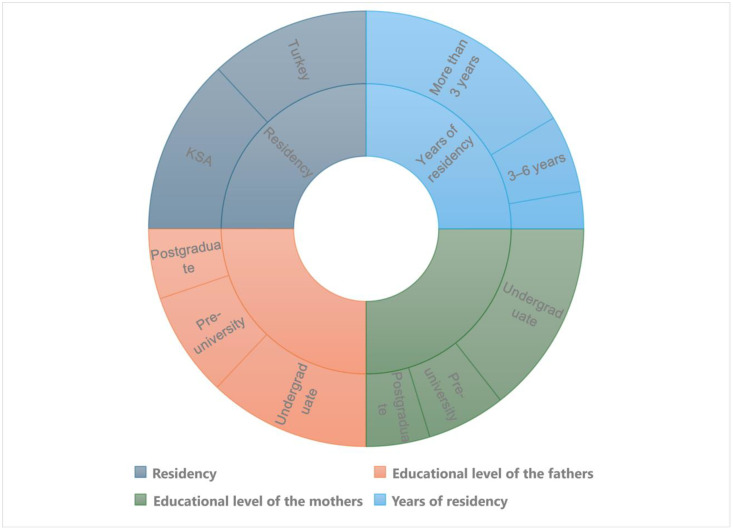
Demographic distribution of participants by residency, parental education level, and years of residency.

Table 1, which presents the participant demographics of 418 participants, reveals that slightly more participants lived in Saudi Arabia (52.4%) than Turkey (47.6%), most fathers (48.1%) and mothers (57.7%) had an undergraduate degree, and most participants had lived in the host country for longer than 6 years (66%). The participants’ demographics are presented visually in Fig 1.

### Instruments

An online structured questionnaire developed by the researcher containing two sections was administered to the participants to identify the challenges faced by refugee children in Saudi Arabia and Turkey. It was constructed based on the theoretical framework proposed by Vygotsky [39], findings of prior studies [4,15,35,45], relevant educational literature addressing the challenges faced by individuals residing outside their home countries, and expert insights from the field. The questionnaire was developed in the Arabic and Turkish languages to ensure appropriateness for the participants. It was also composed of closed-ended questions to ensure that it directly addresses the research.

The first section of the questionnaire was related to the participants’ demographic information, including (1) type of parent (mother or father), (2) refugee residency status (Saudi Arabia or Turkey), (3) parental education level (pre-university [high school or other], undergraduate [bachelor’s degree], or postgraduate [master’s or doctoral program]), and (4) years of residency (less than 1 year to less than 3 years; 3–6 years; more than 6 years). The second section encompassed three parts, each comprising ten statements related to the (1) educational challenges (such as those concerning communication with teachers, linguistic readiness, curricula and study program), (2) social challenges (such as lack of friends and companions, lack of awareness about customs and traditions, differences in timing of social events), and (3) cultural challenges (such as lack of cultural resources, lack of freedom to practice their cultural and intellectual customs, weak awareness of cultural heritage) that refugee children face. The full questionnaire is provided in S1 Appendix.

### Validity and reliability of the instrument

The external validity of the instrument was ensured by conducting a detailed cross-examination of the questionnaire with a group of 12 experts in education, administration, planning, comparative studies, and psychology. The experts reviewed the topic, questions, and objectives of the study and provided feedback on the suitability of the questionnaire for the study topic, its potential to reveal targeted information, coherence of each statement with its corresponding dimensions, clarity of the statements, and suggestions for improvement. Based on the experts’ feedback, some statements were modified, and additions and deletions were made to ensure the questionnaire was suitable for the study. This study was approved by the Human Research Ethics Board at King Saud University (IRBKSU-HE-24-180).

After assessing its external validation, the questionnaire was administered to 100 parents as an exploratory sample. The internal consistency of the statements was assessed using Pearson’s correlation coefficient between each statement and the total questionnaire score. The correlation coefficients for all the questionnaire statements were positive, ranging from 0.541 to 0.869. The correlation coefficients for all the questionnaire sections were strongly positive, ranging from 0.769 to 0.932, and all were significant at the 1% level. These results confirmed the validity of the questionnaire and its items.

The reliability of the questionnaire was calculated using Cronbach’s alpha coefficient. The results are presented in Table 2 and indicate high reliability for all questionnaire sections. All Cronbach’s alpha coefficients for the questionnaire sections were high, ranging from 0.802–0.844. The Cronbach’s alpha coefficient for the complete questionnaire was 0.911, indicating its reliability and supporting its potential to yield consistent results in the present study.

**Table 2:** The results of the questionnaire’s reliability calculated using Cronbach’s alpha.

Dimension	Number of statements	Cronbach’s alpha	Degree of reliability
First (Educational challenges)	10	0.844	High
Second (Social challenges)	10	0.802	High
Third (Cultural challenges)	10	0.823	High
Total	30	0.911	High

Responses were recorded on a **3-point Likert scale** ranging from 1 (**High Approval**) to 3 (**Low Approval**). Higher scores indicated greater approval of the perceived challenges in that domain. These scores were then multiplied by their corresponding frequency, summed, and divided by the total sample size to derive the weighted mean. The weighted mean served as a metric to represent the relative significance of each statement in the dataset. The level of agreement within the study sample was quantified using the following formula:


𝐀𝐠𝐫𝐞𝐞𝐦𝐞𝐧𝐭 𝐥𝐞𝐯𝐞𝐥 = (𝐧−1)/𝐧,

where (n) denotes the number of responses, equivalent to three options in this context.

### Statistical methods

The researchers used various statistical methods to conduct descriptive and inferential analyses of the questionnaire statements. Descriptive analysis involved calculating the percentages for frequencies, determining the mean values, assessing the relative weights, and calculating the standard deviations to answer the first three research questions. However, t-tests for two independent samples and t- and p-values were used to determine the differences in the participants’ perspectives according to the country of residency and parents’ educational level to answer the fourth and fifth research questions.

## Results

Below, the findings have been systematically organized according to the research questions. That is, each section corresponds to a research question, accompanied by a structured empirical response, to enhance clarity and coherence, thus facilitating a nuanced understanding of how each aspect of the study aligns with its associated research objective.

### RQ1: What are the educational challenges faced by refugee children residing in Saudi Arabia and Turkey?

For this question, the overall mean of the statements in the first section was (M = 1.8249), demonstrating medium agreement. Across all statements in the first section, the values varied between (M = 1.5574, SD = 0.606) and (M = 2.0144, SD = 0.794) on a scale of 3. Table 3 lists the descriptive statistics of the educational challenges and highlights the ongoing efforts of both Saudi Arabia and Turkey to incorporate and assimilate refugee children into their educational institutions alongside their citizens.

**Table 3:** Descriptive statistics of the educational challenges faced by refugee children residing in Saudi Arabia and Turkey.

No.	Statements	Mean	SD	Rank	Level of agreement
7	Insufficient number of peers for refugee children to collaborate with in the educational field	2.0144	0.7262	1	Moderate
4	Differences in the curricula and study programs from what the refugee children are accustomed to	2.0096	0.6994	2	Moderate
3	Lack of linguistic readiness	1.9833	0.7940	3	Moderate
9	Refugee children’s perception of a lack of objectivity in interactions with children from the native community in the school	1.9761	0.7260	4	Moderate
6	Absence of a familiar educational environment for refugee children within the local community	1.9522	0.7666	5	Moderate
1	Difficulty enrolling at government schools	1.8517	0.7848	6	Moderate
2	Limited communication with teachers within the school	1.7584	0.7404	7	Moderate
10	Absence of equal educational opportunities for refugee children as for children of the native community	1.5766	0.6386	8	Low
8	Weak ability to compete educationally with children of the native community	1.5694	0.6356	9	Low
5	Difficulty in refugee children’s adaptation to evaluation systems in schools	1.5574	0.6060	10	Low
Total		1.8249	0.4344		Moderate

The responses indicated that the statements with the highest levels of agreement in the section were ranked first, second, and third, as represented in the questionnaire by statements (7), (4), and (3), respectively (S1 Appendix). These statements highlighted the challenges related to a lack of peer collaboration in the educational field, differences in the curricula and educational programs from what the refugee children were accustomed to, and a perceived deficiency in language readiness.

The relative weights assigned to these statements were (2.0144), (2.0096), and (1.9833), respectively, indicating moderate levels of significance. By contrast, statements with the lowest levels of agreement were ranked lower, as represented in the questionnaire by statements (5), (8), and (10) (S1 Appendix). These statements addressed challenges, such as poor adaptation to the school evaluation systems and perceived weak ability to compete educationally with local children. They also addressed the perceived lack of educational opportunities equal to those available to children that are native to the community. The relative weights assigned to these statements were (1.5574), (1.5694), and (1.5766), respectively, indicating low levels of significance.

### RQ2: What social challenges do refugee children residing in Saudi Arabia and Turkey encounter?

The means and standard deviations of statements related to social challenges were calculated for this question. Table 4 shows that the mean score for statements related to the second section was 2.4179, signifying a general “high” level of agreement. The mean values for the statements ranged from 2.2871 to 2.6005, with a possible range of 3. In addition, the standard deviations varied from 0.5718 to 0.7591. These findings can be understood in the context of the different social contexts and cultural values in both countries, posing challenges for refugee children to adapt to and integrate into these conditions. This is particularly noteworthy, as the beliefs and overall behaviors of refugee children may be influenced, affecting their creative skills and interactions within the community and, in later life, at the workplace.

**Table 4:** Descriptive statistics for the social challenges faced by refugee children residing in Saudi Arabia and Turkey.

No.	Statements	Mean	SD	Rank	Agreement level
8	Loss of trust in others	2.6005	0.5963	1	High
9	Weak sense of social safety	2.5287	0.6041	2	High
7	Feeling of social isolation	2.5215	0.6353	3	High
1	Lack of friends and companions	2.4809	0.5718	4	High
5	Low level of adaptation to the food and drink in the host nation	2.4163	0.7157	5	High
3	Lack of awareness of societal customs and traditions in the host nation	2.4139	0.6298	6	High
10	Difference in the timing of social events that they were accustomed to in the original society	2.3158	0.7591	7	Moderate
4	Difference in the dressing style from what they were accustomed to in the original society	2.3086	0.5736	8	Moderate
6	Difficulty in accessing entertainment opportunities that they were accustomed to in the original society	2.3062	0.5728	9	Moderate
2	Weak adaptation to social events	2.2871	0.6952	10	Moderate
Total		2.4179	0.4499		Moderate

Moreover, the evident sense of detachment experienced by the refugee children highlights their perception of weak connections with their surroundings. Consequently, they may become less concerned about the opinions, traditions, and beliefs of their original society and display a sense of rebellion and non-conformity. Regarding the statement rankings, the top-ranking statements with the highest responses in the section were (8), (9), and (7), addressing the loss of trust in others, weak sense of social safety, and feelings of social isolation, with relative weights of (2.6005), (2.5287), and (2.5215), respectively, all classified as high.

Conversely, statements (2), (6), and (4) received the lowest responses, indicating a low level of adaptation to social events, difficulty in accessing entertainment opportunities customary in the original society, and a difference in the dressing style from what was customary in the original society. The mean scores for these statements were (2.2871), (2.3062), and (2.3086), respectively, all categorized as moderate.

### RQ3: What cultural challenges do refugee children in Saudi Arabia and Turkey face?

Table 5 illustrates that the overall mean for the statements related to the third section was 2.4313 out of 3, indicating a high level of agreement. The mean for the statements ranged between 2.2919 and 2.5574, while the standard deviations ranged between 0.602 and 0.7187.

**Table 5:** Descriptive statistics for the cultural challenges faced by refugee children residing in Saudi Arabia and Turkey.

No.	Statements	Mean	SD	Rank	Agreement level
3	Refugee children suffer from the bias of the members of the host society towards their culture	2.5574	0.6020	1	High
5	Weak ability to highlight their cultural and intellectual heritage and take pride in it in front of members of the host society	2.5191	0.6649	2	High
4	Absence of permission to practice their cultural and intellectual customs in the host society	2.5144	0.6355	3	High
8	Negligence of educational programs that incorporate the intellectual and cultural heritage of refugee children into the host society	2.4593	0.6639	4	High
1	Mental disturbance because of the differences between the cultures of the original and host societies	2.4569	0.6674	5	High
7	Scarcity of resources that can connect the refugee children to their cultural heritage in the host society	2.4545	0.6672	6	High
10	Contradiction between the intellectual and cultural heritage of refugee children and that of the host society	2.4115	0.6592	7	High
9	Weak awareness of the intellectual and cultural heritage of the host society	2.3254	0.6111	8	Moderate
2	Lack of resources for cultural communication	2.3230	0.7187	9	Moderate
6	Difficulty of engaging in positive interactions with individuals of different cultures with varied beliefs	2.2919	0.6317	10	Moderate
Total		2.4313	0.5051		High

The highest-ranked statements were (3), (5), and (4), each of which addressed a different aspect of the children’s experiences. Statement (3) highlighted children’s suffering from the bias exhibited by members of the original society towards their culture, while statement (5) underscored their weak performance in emphasizing their cultural and intellectual heritage. Statement (4) focused on the absence of permission to practice their cultural and intellectual customs. These statements were categorized as “high” with relative weights of (2.5574), (2.5191), and (2.5144), respectively.

By contrast, statements (6), (2), and (9) received the lowest responses, indicating a low ranking. These statements addressed challenges related to positive interactions with those who had rebellious thoughts and belonged to different cultures, lacked robust resources for cultural communication, and had a weak awareness of the intellectual and cultural heritage of the society to which they had relocated. The relative weights for these statements were (2.2919), (2.323), and (2.3254), respectively, all falling under the “moderate” category.

### RQ4: How do the challenges encountered by refugee children vary by their country of residence (Saudi Arabia and Turkey), and are they statistically significant?

The results revealed no significant differences based on the t- and p-values analysis for the three sections (educational, social, and cultural challenges, see Table 6). No statistically significant difference was found between refugee parents in Saudi Arabia and Turkey in terms of their children’s educational [t(418) = 1.66, p = 0.097], social [t(418) = −0.81, p = 0.418], and cultural challenges [t(418) = −1.176, p = 0.24]. The findings indicated that the participants in both countries had similar perspectives on the challenges faced by refugee children, which could be attributable to shared agreements and attitudes towards refugees in both countries.

**Table 6:** Challenges encountered by refugee children according to the country of residence: Turkey and Saudi Arabia.

Section	Country of residence	Number	Mean	SD	t-value	p-value
First (Educational challenges)	Saudi Arabia	219	18.58	3.92	1.661	0.097
	Turkey	199	17.88	4.75		
Second (Social challenges)	Saudi Arabia	219	21.72	3.83	−0.810	0.418
	Turkey	199	22.04	4.33		
Third (Cultural challenges)	Saudi Arabia	219	24.04	5.35	−1.176	0.240
	Turkey	199	24.62	4.70		

**Fig 2:**
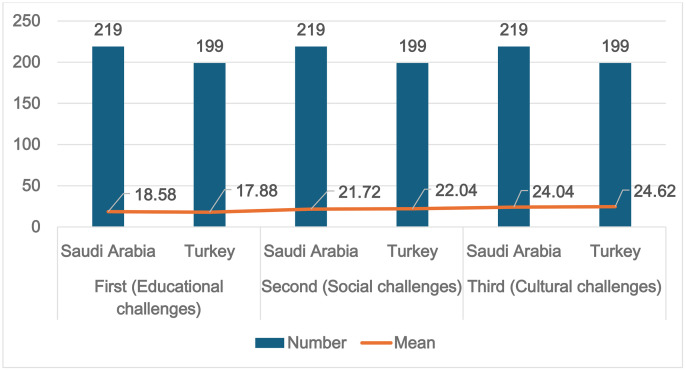
Challenges faced in Saudi Arabia and Turkey: Frequencies and mean scores.

Fig 2 illustrates the sample sizes (219 from Saudi Arabia and 199 from Turkey) and mean scores for each of the three categories of challenges. For both countries, educational challenges showed the lowest mean scores (Saudi Arabia: 18.58, Turkey: 17.88), while cultural challenges showed the highest (Saudi Arabia: 24.04, Turkey: 24.62). This suggests that participants in both countries experienced cultural challenges with greater intensity than they did educational or social challenges. In other words, no significant cultural or religious differences were found, as the refugee groups in both countries practiced Islam. Furthermore, refugee children were accustomed to and familiar with common and well-known traditions and Islamic celebrations, such as Eid al-Fitr, Eid al-Adha, and Ramadhan.

### RQ5: How do refugee parents’ perspectives (in both countries) statistically differ in terms of the challenges their children faced based on their education level?

**Fig 3:**
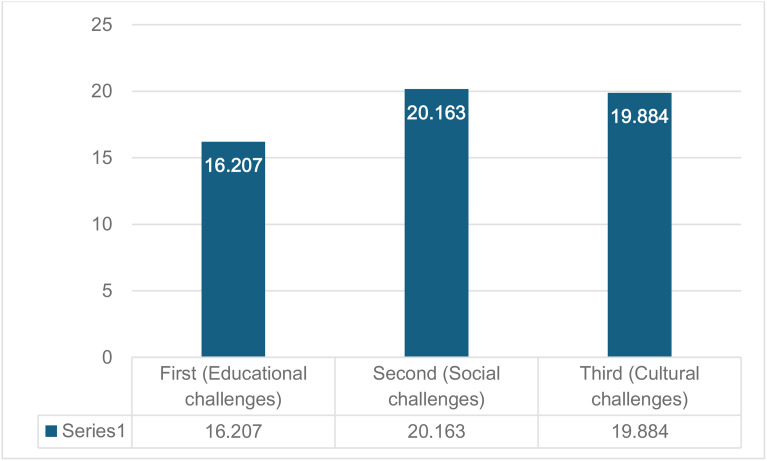
Comparison of f-statistics across challenge domains.

Responses to the fifth question revealed statistically significant differences in the challenges faced by refugee children in Saudi Arabia and Turkey, influenced by parental education level (pre-university/undergraduate/postgraduate). Table 7 illustrates the differences in the mean responses of the participants, highlighting statistically significant differences in the challenges faced by refugee children in both countries based on parental education level, with F-values of (16.207), (20.163), and (19.884) for the first (educational), second (social), and third (cultural) sections, respectively. All these values were statistically significant at the 5% level, emphasizing the substantial differences in the perceptions of parents with diverse educational backgrounds and providing nuanced insights into the experiences of refugee children in Saudi Arabia and Turkey. The lines in Fig 3 visually depict this data.

**Table 7:** Differences in challenges according to parental educational level.

Section	Source of differences	Sum of squares	F	Mean squares	F-value	p-value
First (Educational challenges)	Between groups	570.03	2	285.013	16.207	0.0001
	Within the group	7298.10	415	17.586		
	Total	7868.12	418			
Second (Social challenges)	Between groups	612.61	2	306.303	20.163	0.0001
	Within the group	6304.42	415	15.191		
	Total	6917.02	418			
Third (Cultural challenges)	Between groups	930.43	2	465.217	19.884	0.0001
	Within the group	9709.51	415	23.396		
	Total	10639.94	418			

**Fig 4:**
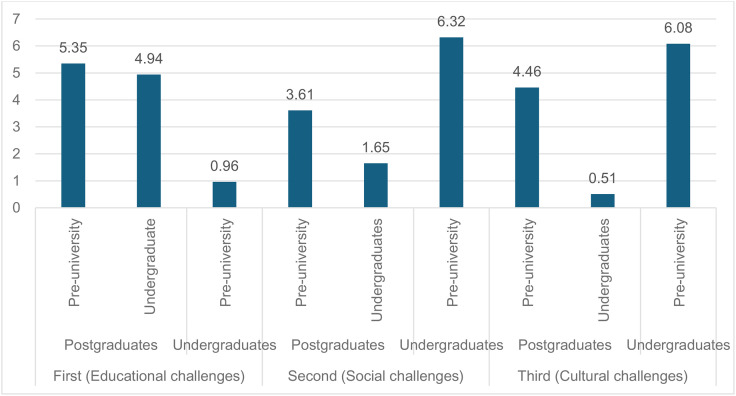
Comparison of *t‑*statistics for challenge.

Fisher’s least significant difference (LSD) pairwise comparison test was used to assess differences in the three survey sections based on the father’s education level (see Table 8 for more details). Statistically significant differences were identified in the responses concerning the educational challenges, favoring participants with postgraduate qualifications over those with pre-university (3.10, p = 0.00) or undergraduate (2.65, p = 0.00) qualifications. Additionally, significant differences were observed in the social and cultural challenges, favoring participants with university qualifications over those with pre-university qualifications (2.77, p = 0.00 for social; 3.30, p = 0.00 for cultural). Notably, no statistically significant differences were found between participants with postgraduate and university qualifications regarding social (0.82, p = 0.10) and cultural (0.31, p = 0.61) challenges, and no differences were observed between participants with university and pre-university qualifications regarding educational challenges (0.45, p = 0.34). The results of the statistical analysis are presented in Fig 4.

**Table 8:** Differences in the challenges faced by refugee children according to their fathers’ educational levels.

Section	Group A	Group B	Differences in Means between A and B	Standard error	p-value
First (Educational challenges)	Postgraduates	Pre-university	3.10964^*^	0.581	0.0001
		Undergraduate	2.65843^*^	0.538	0.0001
	Undergraduates	Pre-university	0.45121	0.472	0.340
Second (Social challenges)	Postgraduates	Pre-university	1.94969^*^	0.540	0.0001
		Undergraduates	−0.82501	0.500	0.100
	Undergraduates	Pre-university	2.77470^*^	0.439	0.0001
Third (Cultural challenges)	Postgraduates	Pre-university	2.99195^*^	0.670	0.0001
		Undergraduates	−0.31601	0.621	0.611
	Undergraduates	Pre-university	3.30796^*^	0.544	0.0001

**Fig 5:**
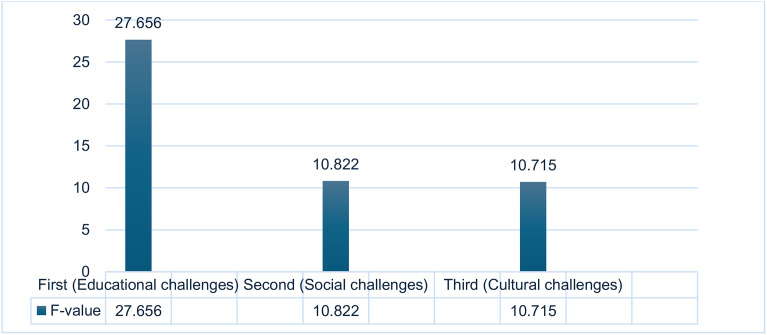
Comparison of f-values across educational, social, and cultural challenges.

The findings also revealed significant differences in the severity of challenges based on the mother’s education level. Participants’ responses demonstrated statistically significant variations observed from the parent’s perspective, specifically that of the mother. The significance values (F) for the three survey sections were (27.656), (10.822), and (10.715), respectively. Differences were considered statistically significant at a 5% significance level (see Table 9 for further details). Fig 5 below shows a visual representation of the data attained.

**Table 9:** Differences in challenges faced by refugee children according to their mothers’ educational levels.

Section	Source of differences	Sum of Squares	DF	Mean of squares	F-value	p-value
First (Educational challenges)	Between groups	925.34	2	462.668	27.656	0.0001
	Within groups	6942.79	415	16.730		
	Total	7868.12	418			
Second (Social challenges)	Between groups	342.87	2	171.433	10.822	0.0001
	Within groups	6574.16	415	15.841		
	Total	6917.02	418			
Third (Cultural challenges)	Between groups	522.43	2	261.216	10.715	0.0001
	Within groups	10117.51	415	24.380		
	Total	10639.94	418			

The LSD pairwise comparison test was used to examine differences in the severity of challenges in the responses across the three survey sections (educational, social, and cultural challenges) based on the mothers’ education levels. Results revealed statistically significant differences in the severity of challenges for the first section, favoring those with postgraduate qualifications over those with pre-university and university qualifications. Differences were considered statistically significant at the 5% level. Additionally, significant differences were found across all three sections based on the mother’s education level, favoring those with university qualifications over those with pre-university qualifications. However, no significant differences were identified between participants with postgraduate and university qualifications in their responses regarding the social and cultural challenges. Table 10 illustrates the differences between the participants with postgraduate and university qualifications.

**Table 10:** LSD comparative values regarding the differences between participants with postgraduate and university qualifications.

Section	Group A	Group B	Differences between Means of A and B		Standard error
First (Educational challenges)	Postgraduates	Pre-university	4.24038^*^	0.618	0.000
		Undergraduates	1.18131^*^	0.530	0.026
	Undergraduates	Pre-university	3.05907^*^	0.490	0.000
Second (Social challenges)	Postgraduates	Pre-university	2.09442^*^	0.602	0.001
		Undergraduates	−05678.0	0.516	0.912
	Undergraduates	Pre-university	2.15120^*^	0.477	0.000
Third (Cultural challenges)	Postgraduates	Pre-university	3.16675^*^	0.747	0.000
		Undergraduates	.847210	0.640	0.186
	Undergraduates	Pre-university	2.31954^*^	0.592	0.000

## Discussion and conclusion

The findings of this study reveal important insights into the educational, social, and cultural challenges faced by refugee children in Saudi Arabia and Turkey. These results can be understood in the context of the different social environments and cultural values in both countries, which lead to variations in interests and cultural practices, thus posing significant challenges to refugee children’s adaptation to and integration into these host societies [7,20]. These challenges affect their behavior, beliefs, and interactions, potentially shaping their creative development and participation in community settings.

Specifically, this study found that refugee children encounter moderate challenges in the educational domain and that these challenges can be attributed to the inherent differences in educational systems, curricula, and programs. These finding are consistent with those of Al-Mansour [15], who reported that refugee children face several challenges when integrating into the education system in Saudi Arabia. Further, they are consistent with those of both Al-Mansour [15] and St. Nicholas [30], who revealed that the difficulties faced by refugee students were due to differences in the nature of the curricula and a lack of linguistic readiness [4]. By contrast, Caliskan’s [13] study indicated that school principals worked diligently to ensure a socially equitable environment for children. However, as Kus-Harbord and Ward [37] have emphasized, refugee acculturation and educational integration are shaped not only by school-level efforts, but also by the broader sociopolitical context of the host country. Therefore, while educational institutions play a central role in supporting refugee learners, sustained progress also depends on inclusive policies and supportive environments at the national and community levels.

Moreover, consistent with the UNHCR [2], the findings from the current study confirm that the complex and multifaceted challenges that refugee children face, which are related to the disruption of their prior educational experiences, the psychological effects of forced migration, and the need to adapt to new and often unfamiliar educational systems, contribute to social withdrawal, isolation, and the emergence of behavioral problems among this group [17,46]. This indicates that the difficulties refugee children face are not limited to academic aspects, but also encompass social and emotional dimensions, as discussed below, reflecting the broader consequences of displacement and cultural disintegration [32].

Nevertheless, regarding parents’ perspective on their children’s education-related challenges, the data illustrate differences in perspectives between parents with postgraduate education and those possessing undergraduate qualifications. The findings underscore the effect of parental education level on the perceived educational and social hurdles that refugee children in Saudi Arabia and Turkey contend with, favoring parents with higher academic levels. This phenomenon can be ascribed to highly educated parents’ enhanced comprehension of education-related and social challenges. This perception emanates from their accumulated experience, exposure to real-world situations, and firsthand encounters with the identified challenges.

In addition to educational challenges, the present study identified the following social factors that impede the integration of refugee children into the host society: lack of trust, weak sense of safety, social isolation, lack of friends and companions, lack of adaptation to local food and drink, and lack of awareness of local societal customs and traditions. In particular, the data in this study showed that the social challenges faced by refugee children in their host countries were moderate, with loss of trust in others and a weak sense of social security ranked among factors exerting the most impact. This indicates a notable decline in social trust among these children. Feelings of social isolation and the absence of friends and peers also emerged as some of the most significant challenges, reflecting the difficulties refugee children face in forming meaningful social relationships. These findings partially align with those of a previous study by Acar-Ciftci [16], who noted the prevalence of racial discrimination, occasionally experienced in educational circles, wherein refugee children are considered foreigners unequal to local children, leading to frustration, disappointment, social isolation, and even poor academic performance. These results also align with those of Caliskan [13], who examined the marginalization that refugee students may encounter in Turkish schools and affirmed that social challenges have a substantial impact on refugees. This perspective has significant implications for education, as it suggests a focus on collaborative learning and recognizes the role of the social context in shaping the understanding of refugee children’s situations [39].

Further, the enrolment rates between local (Turkish) and refugee (Syrian) children significantly differed due to differences in not just economic characteristics, but in social factors as well. This finding aligns with the results of Kırdar et al. [4], who have indicated that both social and economic factors explain a large part of the gap in school enrollment rates between refugee children and Turkish children. This finding is also consistent with the study by Shuayb et al. [6], which showed that social and economic barriers continue to hinder refugee education, even in supportive contexts. Therefore, enrolling refugee children in host country schools without addressing these barriers limits their full integration, as local systems often cannot meet their specific needs.

Refugee children were also found to face language barriers and have significant household responsibilities, factors that possibly hindered their continued participation in the formal education system [5]. This was also confirmed by Celik et al. [12], who indicated that, besides challenges such as restricted access to the educational system [13] and cultural adaptation difficulties, refugee children also face challenges in language learning. Sarmini et al. [47] also pointed out that refugee children in Turkey struggle with the local language, which poses a barrier to effectively communicating in society.

Concerning cultural integration, the present study also found that refugee children in both Turkey and Saudi Arabia face challenges in attaining cultural acceptance due to differences in cultural practices, norms, and resources available to them for integration into the host society. However, the perspectives of the parents from both countries concerning their children’s cultural challenges were similar. This was likely due to their shared Islamic culture and attitudes, and the fact that refugee children were accustomed to and familiar with common and well-known traditions and Islamic celebrations, such as Eid al-Fitr, Eid al-Adha, and Ramadhan. Thus, differences in cultural or religious challenges were not significant.

Additionally, this study found no statistically significant differences in the parents’ perceptions of cultural challenges according to their education level. That is, parental education level had no statistically significant impact on the parents’ perception of the cultural challenges their children face, which is likely attributable to the universal nature of the cultural challenges encountered by the parents of refugee children, irrespective of their educational backgrounds.

From the perspective of the social-cultural theory, these findings confirm that the interconnectedness of refugee children’s development and the impact of integrating the original and new cultural environment aspects may affect the way they integrate into society [48] because learning and cognition are not merely an individual process but rather deeply rooted in social and cultural contexts. Therefore, social integration through sustainable education for refugee children is a key component in addressing the educational, social, and cultural needs of one of the world’s most vulnerable populations [32]. By investing in the education of these children, stakeholders can contribute to strengthening social cohesion and supporting flexible cultural integration within refugee communities [14].

Lastly, the findings revealed that family income was not a significant determinant in shaping parents’ understanding, interpretation, or experience of the educational, social, and cultural challenges encountered by their children. Parental responses demonstrated a notable consistency across varying income levels, whether families possessed limited, moderate, or substantial financial resources. That is, concerns related to school integration, peer relationships, and cultural adaptation were expressed with similar intensity regardless of economic status. This means that better financial standing did not appear to confer either heightened or diminished awareness of these challenges. This is consistent with the findings of previous studies that indicated that the movement of individuals from different cultural backgrounds has specific social effects on them and their host countries, regardless of their economic situations [5,26].

The present study is novel in that it explores these factors in the Saudi Arabian context, thereby filling the gap in the extant research. It suggests the importance of addressing the above-described areas of concern to facilitate refugee children’s participation in social and cultural settings and, consequently, their integration into host societies.

### Recommendations

This study’s results highlight the need for government-led initiatives to increase support for refugee children in Saudi Arabia and Turkey. National committees should be set up to regularly assess the circumstances and requirements of refugee children and their families. This will help to monitor their wellbeing, ensure timely interventions, and facilitate informed decision-making and tailored assistance.

First, policies should guarantee equal access to early childhood education for refugee children and institutionalize their rights. Second, the governments should offer language support services and psychosocial care, such as by recruiting bilingual teachers or those with refugee backgrounds, ultimately fostering their social and emotional well-being. Third, legal frameworks, including policies and regulations, should be established or revised to enable refugees to maintain and showcase their cultural and social identities in a way that honors the host country’s laws. For example, municipal governments in both countries could allow refugee communities to hold cultural events or storytelling sessions in public schools, with oversight to ensure respect for local policy, thus promoting a sense of community and belonging. Fourth, establishing joint refugee support units (e.g., through formal cooperation between the ministries of education, health, and social services) in cities with substantial Syrian populations, like Gaziantep in Turkey or Riyadh in Saudi Arabia, could be a highly effective strategy for providing them with comprehensive support, considering their varied requirements, and enhancing their assimilation into the host community. By combining education departments, social workers, and mental health counselors into a single unit, these entities could address the multifaceted needs of refugees in an integrated and well-organized fashion.

In school and community settings, several practices are recommended to guarantee the successful integration and education of refugee children. First, adapting curricula is essential, as it could allow refugee children to relate to the curriculum and experience a sense of belonging in their new surroundings. For example, teachers could incorporate picture books with refugee stories in Arabic and Turkish to encourage a sense of belonging and support language growth among students. This approach can be particularly effective in enabling students to connect with their cultural heritage and cultivate a more robust sense of identity.

Second, schools could introduce culturally sensitive materials that explain daily routines in Arabic dialects that Syrian students are familiar with, enabling them to navigate their new surroundings with increased ease and confidence. This could be achieved by prioritizing cultural and linguistic needs, such as integrating multicultural content and implementing trauma-informed teaching methods.

Third, language readiness programs should be introduced in kindergartens to assist refugee children in acquiring fundamental communication skills in the host country’s language. This is crucial, as it could provide a basis for future academic achievement and social assimilation, enabling refugee children to communicate efficiently with their classmates and instructors, thereby decreasing feelings of loneliness and ostracism.

Fourth, training teachers in cultural competency, trauma sensitivity, and inclusive classroom methods is essential for refugee students to achieve academic success and maintain their emotional well-being. This specialized training would empower educators to establish a supportive learning environment that recognizes the distinct challenges encountered by refugee students. Training for early childhood educators on teaching in multicultural and refugee settings would provide them with the knowledge and skills required to meet the varied needs of refugee students. Further, investing in teacher training can create a more inclusive and equitable learning environment for all students. Fifth, parents of refugee children should be given the opportunity to socialize within and deliver workshops and presentations to their local communities. Refugee parents could share cultural practices such as cooking, crafts, or stories through events hosted by schools, including mini workshops. This could enable culture sharing, foster cross-cultural understanding, and encourage local families to appreciate the diversity within the refugee community.

### Limitations and future research directions

This study has certain limitations. First, the literature review was not extensive owing to a lack of sufficient studies in the field of refugee childhood education in the context of Saudi Arabian education. Second, the data collected from parents, who were answering on behalf of their children, may not accurately represent the children’s real-life experiences or emotions because of the inherent constraints of parental viewpoints. A significant concern is that this potential discrepancy may result in biased or incomplete information, which could influence the conclusions drawn. The self-reported data limits the capacity to authenticate the information’s accuracy, as there is no external confirmation or corroboration to validate the responses. Consequently, the results may be susceptible to mistakes or inaccuracies, and the capacity to make objective comparisons or generalize the outcomes across wider demographics is significantly constrained. Additionally, the generalizability of the study results can be enhanced by including refugees from other cities in Turkey and Saudi Arabia to generate a larger sample size. Future studies might divide the sample based on age groups (e.g., early childhood, childhood, and adolescence) to identify the specific factors that impact refugee children or explore the challenges from the children’s perspectives through interviews rather than a questionnaire.

Despite these limitations, the findings of this study can drive further research in specific areas, such as identifying new approaches to strengthen the role of educational institutions in dealing with the challenges faced by refugee children in Saudi Arabia and Turkey. Additionally, future research could examine the relationship between the challenges faced by refugee children in Saudi Arabia and Turkey and their level of national belonging. These studies should aim to offer insight into new educational visions to address the challenges faced by refugee children in these two nations. Finally, they should focus on ascertaining the level of refugee children’s cultural identity in Saudi Arabia and Turkey. By clarifying the educational, social, and cultural settings of refugee children, future research can provide recommendations to help create a safe, harmonious, inclusive, and integrated environment for refugee children that enables them to succeed and thrive.

This study has several strengths as a quantitative study on refugee children’s challenges in Turkey and Saudi Arabia, especially regarding methodology, as it examined parents’ perspectives, valued their experiences, and identified the challenges their children face in the host country. Another strength of this study is related to its contribution to closing the existing research gap by discussing the potential cultural practices and challenges faced by refugee children in Saudi Arabia and Turkey. The findings have important implications for international and local bodies and organizations responsible for assisting refugees. They can also aid in improving the learning outcomes of refugee children and reducing the cultural and social shocks they face by understanding their perspectives and identifying their challenges.

## Supporting information

S1 Appendix Parents Questionnaire. (DOCX)

S1 File Graphical abstract. (DOCX)
