# Supplementary material for: Beyond borders: Exploring the challenges of refugee children in Saudi Arabia and Turkey
Source: PLoS One. 2025 Nov 19;20(11):e0334841. doi: 10.1371/journal.pone.0334841 (PMC12629453; doi:10.1371/journal.pone.0334841)
Supplement: S1 Appendix — (DOCX) [file pone.0334841.s001.docx]

**Appendix**

**S1 Appendix (Questionnaire)**

Dear parents,

We, the researchers, are faculty members at King Saud University and are conducting a study to explore the challenges faced by refugee children in Saudi Arabia and Turkey from their parents’ perspectives.

Please complete the questionnaire survey and indicate your approval (High, Medium, Low) regarding the challenges your children face. The survey comprises two sections: your demographic information and the survey items, which are divided into three axes (educational, social, and cultural challenges).

We assure you that the data will be confidential and used only for scientific research.

We thank you for your valuable time and participation.

Sincerely,

Authors

Check the box if you consent to participating in this survey.

**Part 1: Choose the applicable option**

- **Parent:** Mother; Father

- **Country:** Turkey; Saudi Arabia

- **Educational Level:** Pre-university (High school or other); Undergraduate (Bachelor degree); Postgraduate (Master or Doctoral)

- **Years of Residency:** Less than 1 year to less than 3 years; 3–6 years; More than 3 years

- **Your Child’s Age:** 4–5 years; 5–6 years

| **Statements** | | **Degree of approval** | | |
| --- | --- | --- | --- | --- |
| **The First Axis: Educational Challenges**  What are the educational challenges faced by refugee children residing in Saudi Arabia and Turkey? | | High | Medium | Low |
| 1 | Difficulty enrolling at government schools |  |  |  |
| 2 | Limited communication with teachers within the school |  |  |  |
| 3 | Lack of linguistic readiness |  |  |  |
| 4 | Differences in the curricula and study program from what the refugee children are accustomed to |  |  |  |
| 5 | Difficulty in refugee children’s adaptation to evaluation systems in schools |  |  |  |
| 6 | Absence of a familiar educational environment for refugee children within the local community |  |  |  |
| 7 | Insufficient number of peers for refugee children to collaborate with in the educational field |  |  |  |
| 8 | Weak ability to educationally compete with children of the native community |  |  |  |
| 9 | Refugee children’s perception of a lack of objectivity in interactions with children from the native community in the school |  |  |  |
| 10 | Absence of equal educational opportunities for refugee children as for children of the native community |  |  |  |
| **The Second Axis: Social Challenges**  What social challenges do refugee children residing in Saudi Arabia and Turkey encounter? | | | | |
| 1 | Refugee children’s lack of friends and companions |  |  |  |
| 2 | Weak adaptation to social events |  |  |  |
| 3 | Lack of awareness of societal customs and traditions in the host nation |  |  |  |
| 4 | Differences in the dressing style from what they were accustomed to in the original society |  |  |  |
| 5 | Refugee children’s low level of adaptation to the food and drink in the host nation |  |  |  |
| 6 | Difficulty in accessing entertainment opportunities that they were accustomed to in the original society |  |  |  |
| 7 | Feeling of social isolation |  |  |  |
| 8 | Loss of trust in others |  |  |  |
| 9 | Weak sense of social safety |  |  |  |
| 10 | Difference in the timing of social events that they were accustomed to in the original society |  |  |  |
| **The Third Axis: Cultural Challenges**  What cultural challenges do refugee children in Saudi Arabia and Turkey face? | | | | |
| 1 | Mental disturbance because of the differences between the cultures of the original and host societies |  |  |  |
| 2 | Lack of resources for cultural communication |  |  |  |
| 3 | Refugee children suffer from bias of the members of the host society towards their culture |  |  |  |
| 4 | Absence of permission to practice their cultural and intellectual customs in the host society |  |  |  |
| 5 | Weak ability to highlight their cultural and intellectual heritage and take pride in it in front of members of the host society |  |  |  |
| 6 | Difficulty of engaging in positive interactions with individuals of different cultures with varied beliefs |  |  |  |
| 7 | Scarcity of resources that can connect the refugee children to their cultural heritage in the host society |  |  |  |
| 8 | Negligence of educational programs that incorporate the intellectual and cultural heritage of refugee children into the host society |  |  |  |
| 9 | Weak awareness of the intellectual and cultural heritage of the host society |  |  |  |
| 10 | Contradiction between the intellectual and cultural heritage of refugee children and that of the host society |  |  |  |
