# Supplementary figures and images for: Beyond borders: Exploring the challenges of refugee children in Saudi Arabia and Turkey
Source: PLoS One. 2025 Nov 19;20(11):e0334841. doi: 10.1371/journal.pone.0334841 (PMC12629453; doi:10.1371/journal.pone.0334841)

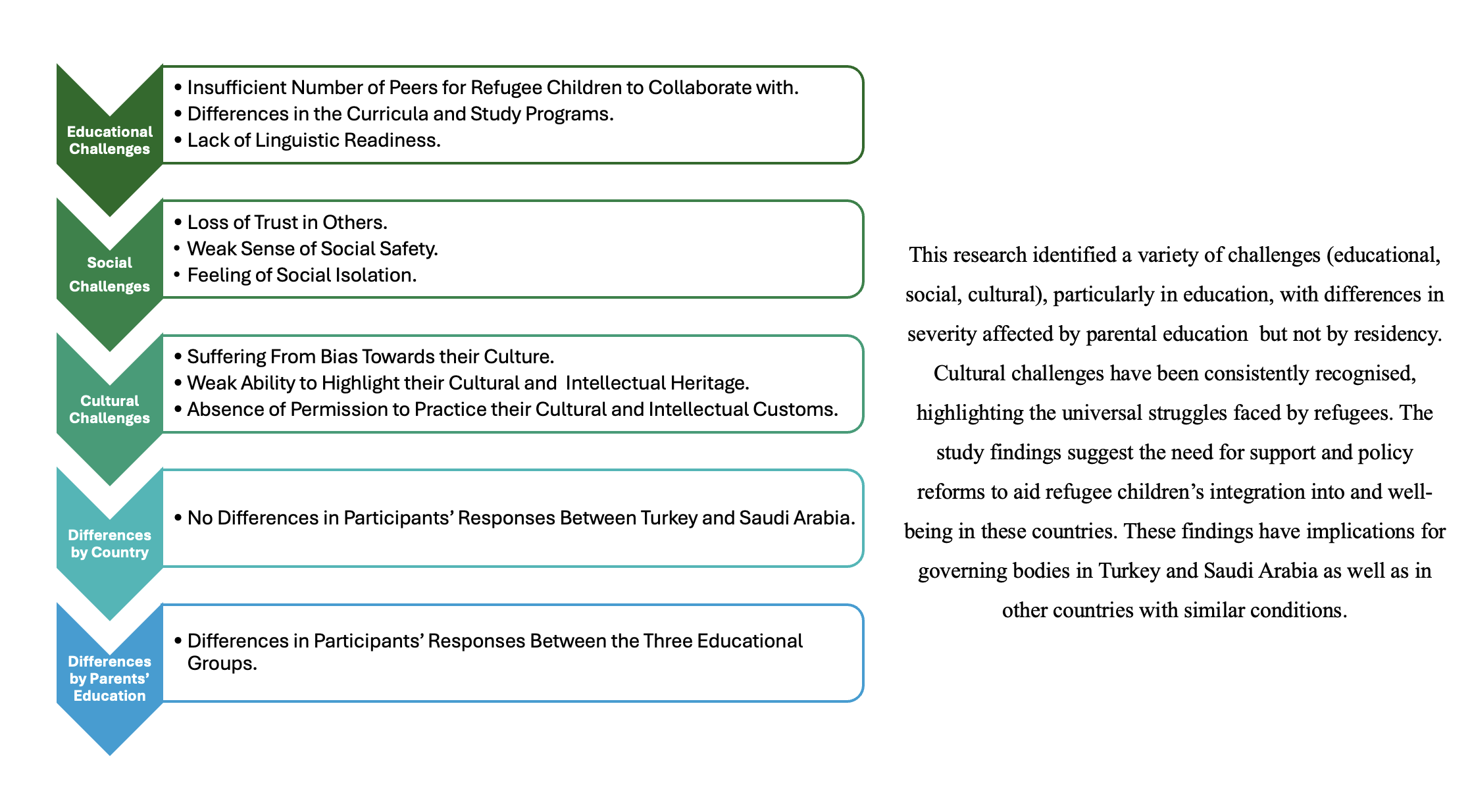


Graphical abstract

Supplement: S1 File — (DOCX) [file pone.0334841.s002.docx]
