# Supplementary material for: Beyond borders: Exploring the challenges of refugee children in Saudi Arabia and Turkey
Source: PLoS One. 2025 Nov 19;20(11):e0334841. doi: 10.1371/journal.pone.0334841 (PMC12629453; doi:10.1371/journal.pone.0334841)
Supplement: Attachment — Submitted filename: PlosOne Article Review.docx [file pone.0334841.s003.docx]

Please note that #Number refers to the line number in the PDF document that I have received .

\# 32 Please Use the most recent UNHCR statistics on the refugees and the proportion of refugees that are children

#32-37. this requires further explanation - more is required here introducing the topic of refugee education - among children?, youth?.

#34 sentence referring to *refugee communities* should introduce this topic. More is required about how these communities are affected by host societies and then an expansion on refugee children’s education.

#38 to #48, references are required and a clearer explanation of plural societies.

#49-50 - *In addition to the economic consequences, various issues related to human rights,*

*democracy, citizenship, and education have emerged [3].* what is this sentence introducing?

The introduction requires a greater focus on the challenges of refugees in plural societies and the issue of educating refugee children.

Literature review - #74 "*refugee children face several challenges*..."only one challenge was mentioned, please expand on this topic.

#75. Please clarify how Job opportunities and social status is relevant to refugee children or omit this sentence.

Please expand on the enrolment rates of refugee children.

#86 *The Kingdom of Saudi Arabia and Turkey are among the Middle Eastern countries that*

*welcome the most refugees*. in relation to what other nations? Please clarify or change the wording of this sentence.

The % of refugees in Turkey have been quoted twice between #85 and #102 and they are different. Please clarify or connect these two statistics in some way. Are there similar statistics available for Saudi Arabia on school age children.

You describe Saudi Arabia and Turkey as diverse nations, more information needs to be included on their diversity and refugee / migrant populations and pathways to these countries.

More is required on education policies or policies related to refugee children from Saudi Arabia.

More is required on the nuanced challenges of refugee children and youth of school-age and their educational experiences related to their refugee status.

Paragraph #107 to #110 requires referencing

#156 - *Aims and Research Questions* - you really need to be specific about the age range of children you are studying. When you refer to *'migrant children'* does this include refugees?

#164-165 - requires a reference. More is required about the similarities and differences between Saudi Arabia so that the reader can understand the context of these two countries in relation to the experiences of refugee children and this information should be consolidated and located in one section in the literature review or the introduction.

More is required about the theory developed by Lev Vygotsky and this should be located in the literature review

#231 – Can you please clarify in this section and in the aims of the research what the age range of the children that are being referred to.

#237 – More is required when explaining the instrument. A sample question and rating scale is required and how the rating scale is interpreted and what does it mean, especially the second section. All demographic variables that were collected should be listed in the section describing the instrument.

#289 – The results section should only state the results of this study in relation to the research questions for the study. Other studies or research reports should be left for the discussion.

#321 – when referring to moderate levels of significance – please clarify whether you mean moderate levels of statistical significance or priority in terms of ranking?

#350-#359; #375-378 should be part of the discussion, please omit from the results section. What should be highlighted here are the similarities and differences between countries in the outcomes of the current study, even though they were not statistically significant, trends should be noted.

Table 8 and Table 9 and possibly Table 10 should be figures to show patterns of scores related to level of parental education.

#472 Discussion – more is required. Every one of the studies’ research questions and outcomes need to be discussed in relation to previous research studies and what the current study’s findings may be adding to the understanding of this issues.

#495 *The social and economic characteristics of Turkish and Syrian Families* –

If data was collected on this issue this should be highlighted in the results section. It should also be discussed in the introduction if there have been previous studies highlighting this issue. In the discussion more is required about this in the discussion – What did Kirdar et al., and Shuayb et al study exactly? And how does the current study add to that understanding and in relation to refugee children.

#498 Recommendations – more is required here. The outcomes of your study have important implications for policies related to refugee children and teaching practices in schools and with families. Government policies and teaching practices should be discussed separately.

You may want to expand your literature review to refugee children of Islamic background in a number of regions and countries that accept immigrants, in order to expand the evidence-base for the consequences of cultural and linguistic differences on the educational experiences of refugee children; and also the shared experience of forced displacement in refugee families that is well documented in research conducted in many other countries.
