# Supplementary material for: Beyond borders: Exploring the challenges of refugee children in Saudi Arabia and Turkey
Source: PLoS One. 2025 Nov 19;20(11):e0334841. doi: 10.1371/journal.pone.0334841 (PMC12629453; doi:10.1371/journal.pone.0334841)
Supplement: Attachment — Submitted filename: REFUGEE_Response_to_Reviewers_.docx [file pone.0334841.s006.docx]

"**Beyond borders: Exploring the challenges of refugee children in Saudi Arabia and Turkey**"

PONE-D-24-51686 (*PLOS ONE*)

Dear Editor,

We addressed the 3 additional comments from the editor Dr Magdalena Szaflarski, during the manuscript resubmission stage.

| few minor edits are requested from the editor | | |
| --- | --- | --- |
| 1 | Lines 185-193: Please clarify that migrant children include refugee children. Also, provide a rationale for the sample ages 4-6 years (lines 192-193). | Thank you for your valuable feedback  We confirm that the term **“migrant children”** in our manuscript also includes **refugee children**.  The present study focused specifically on this stage because, Early childhood particularly the period from four to six years of age—represents a critical stage in the development of personal identity.Thus,targeted early interventions during these formative years can play a pivotal role in fostering a strong sense of belonging and facilitating a smoother, more rapid adaptation to newly encountered communities following migration. |
| 2 | Line 12:  the referent for "their" is unclear. Is it "refugee children's"? Please clarify. | Thank you for pointing out the ambiguity. We have revised the sentence to clarify the referent. The word “their” specifically refers to refugee children’s adaptation to new cultural and social norms…. |
| 3 | Graphical abstract: Please edit the bullet points to all starting with an upper-case letter. Also, last bullet uses a verb structure different from the other bullet points. Please make the form consistent throughout the statements (e.g., no active verb). | Thank you for the helpful feedback. We have updated the graphical abstract bullet points so that:  Each bullet point now begins with an upper-case letter.  The last bullet point has been rephrased to match the noun-phrase style of the other bullets (removing the active verb). |

**Please note that all requested changes are highlighted in gray in the revised manuscript file.**

| Comments to the Authors | | Authors Responses | |
| --- | --- | --- | --- |
| Please ensure that your manuscript meets PLOS ONE's style requirements, including those for file naming. | | Thank you for your comment. We have carefully reviewed PLOS ONE’s style guidelines and ensured that our manuscript now complies with all formatting requirements, including proper file naming conventions.  And we have named both the manuscript and figures files, as well as the tables and appendix within the text according to the journal’s naming style. We have also ensured that paper is double-spaced, kept heading levels to a maximum of 3, and revised the references to follows Vancouver style, among others*.*”  Done ✅ | |
| provide a complete Data Availability Statement in the submission form. | | Since all data underlying this research are provided as part of the manuscript, we have included the following statement in the text under the heading **Data Availability Statement:**  "*All data are in the manuscript and/or supporting information files*"  Done ✅ page 35 | |
| provide ORCID ID | | The ORCID ID in the title page .  Done ✅ | |
| include captions for your Supporting Information files at the end of your manuscript. | | We have listed the following caption of the only supporting file for this paper: “*S1 Appendix. Parents Questionnaire*” At the end of the manuscript after the references list.  Done ✅ Page 41 | |
| **Reviewer #2** | | | |
| **1** | The current graphical abstract is difficult to follow. Choosing a clearer and more structured diagram would improve its accessibility and effectiveness. | Thank you for your feedback. The graphical abstract has been redesigned in a more sequential and clearer format, aligning it with the study’s research questions. It now presents the educational challenges first, followed by the cultural and social challenges, and concludes by highlighting the differences in the participants’ responses.  Done ✅ | Page 2 |
| **2** | One of the key challenges in this study is the comparison between Saudi Arabia and Türkiye regarding refugee children. As noted in lines 86–88, the vast difference in refugee population sizes complicates the interpretation of results. While Türkiye, Lebanon, and Jordan have relatively comparable refugee numbers, the disparity between Saudi Arabia and Türkiye is significant. This should be emphasized in the study, and careful attention must be paid when interpreting the findings. | Thank you for your valuable comment. We acknowledge that the significant difference in refugee population sizes between Saudi Arabia and Turkey poses a challenge for direct comparison of the results. We have highlighted this disparity in the revised manuscript and have been cautious in interpreting the findings accordingly. Also we have expanded the literature review to include studies on refugee children of Arab background in several countries  We appreciate your feedback, which has helped improve the clarity and analysis of the study.  Done ✅ | Page 5-6&7 |
| **3** | The study relies on outdated sources for both countries, with several references exceeding ten years in age. Such references may not accurately reflect the current socio-political context and evolving educational dynamics. To ensure the validity and relevance of the analysis, it is crucial to incorporate more recent research and updated data. | Thank you for your valuable feedback. We acknowledge your concern regarding the old sources that we have used and cited in the paper. To address this, we have reviewed and incorporated the latest research and updated data wherever possible to better reflect the current socio-political context and educational dynamics in both countries.  We have also added around [**27**] recent studies to enhance the content and update the data used, which better represents the current situation in both countries. This has contributed to strengthening the validity and relevance of our analysis. We appreciate your comments, which has helped improve the rigor of the study.  *The recently added studies can be found documented in the reference list at the end of the manuscript  Done ✅ | **The recent added studies:**  1. Androff, D. K. Refugee solutions in the age of global crisis: Human rights, integration, and sustainable development. Oxford University Press ;2022. pp.18–25. doi:[10.1093/oso/9780197642191.001.0001](http://dx.doi.org/10.1093/oso/9780197642191.001.0001).  2. Atalay N, Kılıç Z, Anılan B, Anılan H, Anagun SS. Syrian refugee children’s education in Turkish public schools: primary school teachers’ experiences. ENAD. 2022;22: 265-284. doi: [10.14689/enad.10](https://doi.org/10.14689/enad.10).  3. United Nations High Commissioner for Refugees. Figures at a glance. [Cited June 25, 2025]. Available from: <https://www.unhcr.org/figures-at-a-glance.html>.  8. Mizza D. The pathway to unlocking refugees’ learning potential: current challenges and lifelong learning solutions. In: Wolsey TD, Karkouti IM, editors. Teaching refugees and displaced students: what every educator should know. Cham: Springer; 2023. pp. 139–159. doi: [10.1007/978-3-031-33834-2_11](https://doi.org/10.1007/978-3-031-33834-2_11).#.  10. Fakhry E. Consensus democracy as a framework for accommodating minorities. [Cited March 15, 2023]. Available from: <https://search.mandumah.com/Record/623849>.  11. Celik S, Kardaş İşler N, Saka D. Refugee education in Turkey: barriers and suggested solutions. Pedagog Cult Soc. 2023;31: 687–705. doi: [10.1080/14681366.2021.1947878](https://doi.org/10.1080/14681366.2021.1947878).  13. Jamaludin TS, Nurumal MS, Kyu KW, Noviani W. Sustainable education for refugees’ children: A literature review. Int J Care Scholars. 2024;7: 102-110. doi: [10.31436/ijcs.v7i1.358](https://doi.org/10.31436/ijcs.v7i1.358).  14. Al-Mansour MAH. Culturally responsive education in early childhood. Int J Child Women Stud. 2022;2: 57-77. doi: [10.21608/ijcws.2022.313163](https://doi.org/10.21608/ijcws.2022.313163).  16. Eranıl A, Kasalak G. Yet another crisis? Syrian refugee children and Turkish education system in turbulence. Educ Sci. 2024;49: 97-126. doi: [10.15390/EB.2024.12328](https://doi.org/10.15390/EB.2024.12328).  17. Shah SM, Adam Masumi M. Teaching traditional music in Malaysian schools: considering the cultural context. Int J Learn Teach. 2016;8. doi: [10.18844/ijlt.v8i1.511](https://doi.org/10.18844/ijlt.v8i1.511)  19. General Authority for Statistics. Sustainable development goals (SDGs) indicators report. 2020 [cited 26 June 2025]. Available from: <https://rb.gy/ivq8zd>.  20. Adalı T, Türkyılmaz AS. Demographic data on Syrians in Turkey: what do we know? Int Migr. 2020;58: 196-219. doi: [10.1111/imig.12614](https://doi.org/10.1111/imig.12614).  29. Nimer M. Towards an inclusive education for refugees: a comparative longitudinal study; 2020–2018 education policy analysis in Turkey. Centre for Lebanese Studies. p. 2021. [Cited September 2024]. Available from: <https://lebanesestudies.com/wp-content/uploads/2022/06/Education-Policy-Analysis-in-Turkey.pdf>.  30. European Civil Protection and Humanitarian Aid Operations [Cited 17 July 2024] Available from: <https://civil-protection-humanitarian-aid.ec.europa.eu/where/europe/turkiye_en>  31. Aydin H, Kaya Y. The educational needs of and barriers faced by Syrian refugee students in Turkey: a qualitative case study. Intercult Educ. 2017;28: 456–473. doi: [10.1080/14675986.2017.1336373](https://doi.org/10.1080/14675986.2017.1336373).  32. Çalhan M. Revisiting education resilience of Syrian children in Turkish education ecosystem. Discov Psychol. 2024;4: 182. doi: [10.1007/s44202-024-00297-1](https://doi.org/10.1007/s44202-024-00297-1).  33. Mehri S. The problem of social adaptation of migrant adolescent children. International Journal of Social Science & Interdisciplinary Research ISSN. 2022;11(5): 108–113. https://www.gejournal.net/index.php/IJSSIR.  34. Kim J, Park S-H, Kim M. Stress-related growth associated with acculturation and mental health among international students. J Humanist Psychol. 2025;65: 478–501. doi: [10.1177/0022167820979654](https://doi.org/10.1177/0022167820979654)’  35. Kus-Harbord L, Ward C. Ethnic Russians in post-Soviet Estonia: perceived devaluation, acculturation, well-being, and ethnic attitudes. Int Perspect Psychol. 2015;4: 66–81. doi: [10.1037/ipp0000025](https://doi.org/10.1037/ipp0000025).  38. Chaiklin S. The zone of proximal development in Vygotsky’s analysis of learning and instruction. In: Kozulin A, Gindis B, Ageyev VS, Miller SM, editors. Vygotsky’s educational theory in cultural context. Cambridge University Press; 2003. pp. 39–64. doi: [10.1017/CBO9780511840975.004](https://doi.org/10.1017/CBO9780511840975.004).  39. Moll LC. Vygotsky and education: instructional implications and applications of sociohistorical psychology. Cambridge University Press; 1992.  45. Taha S, Anabtawi M, Al Wreidat T. Silent suffering: the hidden challenges confronting unaccompanied refugee children through the eyes of social workers. Child Abuse Negl. 2024;154: 106868. doi: [10.1016/j.chiabu.2024.106868](https://doi.org/10.1016/j.chiabu.2024.106868).  46. Banks JA. Diversity and citizenship education in multicultural nations. Multicultural Education Review. 2009;1: 1-28. doi: [10.1080/23770031.2009.11102861](https://doi.org/10.1080/23770031.2009.11102861).  47.Palik, J., Østby, G. Interventions to improve refugee children’s access to education and quality learning: A scoping review of existing impact evaluations. Int Rev Educ.2023; 69, 227–247 <https://doi.org/10.1007/s11159-023-10004-2>  48. Pace M, Sen S, editors. Syrian refugee children in the Middle East and Europe: Integrating the young and exiled. Routledge; 2018 Apr 9.  49.Singh M. Pathways to Empowerment: Recognizing the Competences of Syrian Refugees in Egypt, Iraq, Jordan, Lebanon and Turkey. UNESCO Institute for Lifelong Learning. Feldbrunnenstrasse 58, 20148 Hamburg, Germany; 2018. <https://eric.ed.gov/?id=ED599544>. |
|  |  | Thank you for your careful review. We have removed the cited reference [Pinar W, 2010] because it is outdated and no longer reflects the current state of the literature. | **The List of excluded references:**  Pinar W. Curriculum Studies in South Africa: Intellectual Histories and Present Circumstances. Springer; 2010. |
| **4** | statistical information concerning the countries should be drawn from official government documents or legal reports to ensure factual accuracy and reliability. | Thank you for your valuable suggestion. We have ensured that all statistical information concerning the countries is drawn from current official government documents and legal reports such as the United Nations High Commissioner for Refugees (UNHCR) to maintain factual accuracy and reliability throughout the study.  Done ✅ | Page 4- 5&6  For example:  United Nations High Commissioner for Refugees. Figures at a glance. [Cited June 25, 2025]. Available from: <https://www.unhcr.org/figures-at-a-glance.html>  General Authority for Statistics. Sustainable development goals (SDGs) indicators report. 2020 [cited 26 June 2025]. Available from: <https://rb.gy/ivq8zd>. |
| **5** | Certain sentences, such as those in lines 173–174, reflect conceptual inaccuracies by conflating religious sects with ethnic groups, which may lead to confusion regarding the social and religious composition of the population. | Thank you for pointing this out. We have reviewed the sentences in lines 173–174 and corrected the conceptual inaccuracies by clearly distinguishing between religious sects and ethnic groups to avoid any confusion regarding the social and religious composition of the population. Your feedback has helped improve the clarity and accuracy of our discussion.  Done ✅ | Pages:  7-8& 11 |
| **6** | The sample selection process should be explained in greater detail, as the current description lacks sufficient clarity. Without this information, there is a risk of potential bias in the study's findings. | Thank you for highlighting this important issue. We have revised the manuscript to provide a clearer and more detailed explanation of the sample selection process to reduce ambiguity and address potential concerns about bias. Specifically, we have clarified the criteria used for participant inclusion and exclusion, the recruitment method, and the rationale for selecting the study settings.  For example: selected Participants were selected using purposive sampling technique, a type of non-probability sampling to ensure the inclusion of diverse parental perspectives across key demographic categories. The selection criteria included: type of parent…….  Done ✅ | Page 12 -13 |
| **7** | A clearer methodological approach and more detailed explanation are necessary. If the study intended to use a Likert-type questionnaire, this should be explicitly stated. While 'High – Medium – Low' response options may be suitable for assessing intensity or frequency, they are less appropriate for measuring attitudes, beliefs, or satisfaction. In such cases, a standard Likert scale is generally more reliable and widely accepted. | We have added an explanation in the manuscript stating that the participants’ “*Responses were recorded using a 3-point Likert scale ranging from 1 (High Approval) to 3 (Low Approval). Higher scores indicated greater approval perceived challenges in that domain.*” The full questionnaire is provided in Appendix 1.  Done ✅ | Page :16 |
| **8** | Since the paper aims to explore educational, social, and cultural challenges, these topics are addressed in a broad manner. To strengthen the study, the scope should be more narrowly defined, and the research approach to each topic should be clearly articulated. | Thank you for this valuable feedback. We have revised the Introduction and the study objectives to define the scope of the research more narrowly. Specifically, we clarified that the study primarily focuses on educational challenges, in addition to exploring social and cultural challenges faced by refugee children. we also revised the methodology section to explain how each of these dimensions was addressed, both through the design of the questionnaire and the data analysis.  We have also explained the scissions of the questioners give definitions and examples of the three challenges.  Done ✅ | Page :15  The full questionnaire is provided in S1appendex |
| **9** | While the statistical analyses are sufficient to address the stated research questions at a basic level, they are not comprehensive. A more in-depth analysis is needed to enhance understanding and provide deeper insights into the research questions. | Thank you for your valuable observation. In response, we have expanded the statistical analysis section to provide a more in-depth examination of the data. Specifically, we have conducted t-tests for two independent samples and t- and p-values were used to determine the differences in the participants’ perspectives according to the country of residency and parents’ educational level to answer the fourth and fifth research questions. These additions aim to provide a deeper and more nuanced understanding of the patterns and relationships within the data, thereby strengthening the interpretation of the findings.  Done ✅ | Page :17 |
| **10** | to strengthen the overall rigor and clarity of the research, several methodological aspects would benefit from further elaboration. Specifically, greater clarity is needed regarding the criteria and rationale for country selection, the choice of statistical data sources from the literature, the sampling strategy, the explanation of the methodological framework, and the inclusion of more advanced statistical analyses. Addressing these points would enhance the study’s reliability and academic contribution. | We have further clarified our  rationale for selecting the counties we studied (please see the **Scope of the study background** section and the **Participants** subsection of the Method section.)  Done ✅  More details and statistical analysis were added to describe the sample selection strategy  Done ✅ | Page 10- 12-13&15 |
| **Reviewer #4** | | | |
| **11** | Please read again carefully to correct all grammar and translations mistakes. I feel like there are many long and complicated sentence, which makes it difficult to read. | We assure that the manuscript has been checked thoroughly by a professional editor for language, grammar, spelling, structure, coherence, punctuation, and more.in addition of certificate of English editing was provided  Done ✅ |  |
| **12** | Please separate the manuscript into only 4 parts: Introduction, Method, Results, Discussion | Thank you for your suggestion. We have reorganized the manuscript into five main sections, given the importance of the information needed to compare the two countries. These sections include: (1) Introduction, (2) scope and study background, (3) Method, (4) Results, and (5) Discussion and conclusion. We believe this structure enhances the clarity and coherence of the manuscript and better serves the objectives of the study.  Done ✅ |  |
| **13** | ABSTRACT Try to shorten your phrases and be as concise as possible+++ Be more specific about the results. I have trouble understanding this phrase: “The findings revealed various challenges, particularly in education, with differences in severity affected by parental education but not residency.” what severity are you referring too?  You often repeat elements that mean the same thing, seems redundant “universal struggles faced by refugees worldwide” try to simplify either “universal” or “worldwide”.  I don’t understand the meaning of this phrase “findings suggest the need for support and policy reforms to aid refugee children’s integration into (into what?)”  What do you mean by “governing bodies”? Some phrases are complicated and difficult to read and understand “this study expands the existing knowledge base…” simplify+++ for example: “This study contributes significantly to the existing literature on refugee children and the challenges they face, while providing valuable directions for future research” | Thank you for your helpful comment. We have revised the phrasing to make it more concise and specific. Further, we have revised the sentence you pointed out in your comment, ensuring that it is clear. The sentence was meant to indicate that the *severity of educational challenges* varied based on parents’ education levels, but not their residency status, we have now clarified this. We appreciate your feedback as it has helped us improve the Abstract’s clarity.  Done ✅  Thank you for your feedback. We have tightened the structure of our sentences to ensure conciseness and avoid redundancy and wordiness throughout the paper. In the case of this sentence, we have retained only one term “worldwide.”  Done ✅  Thank you for your helpful observations. We have revised the sentence as follows to ensure clarity and completeness: “These findings carry important implications for policymakers in Turkey, Saudi Arabia, and comparable settings, specifically concerning the need for policy reforms and targeted support to facilitate the integration of refugee children into new educational and cultural contexts.”  Done ✅  Thank you for your feedback. We have rephrased the sentences for clarity in the last paragraph of the abstract as per your suggestion.  Done ✅ | Page 3    Page 3  Page 3 |
| **14** | INTRODUCTION “Owing to revolutions….” Is owing the right word? This doesn’t sound correct, you should maybe use the term “due to revolutions” or “as a result of revolutions”  When you cite “26 million refugees” you should give the source.  What do you mean by “type and quality of refugee education depends on the context in which they are accommodated”? for example?  “Given the dimension… in resolving their education related problems” I feel like this phrase is incomplete, what are you trying to say?  Why is the literature review separated from the introduction? It should be included in in or in the discussion.  Overall, the introduction is too long. You don’t have to explain and detail every article. You should write the introduction, make a resume and only put their references. If you want to cite an article you should say “Al-Mansour et al. (18)”  Simplify phrases “to gain insights into the nuanced dynamics of refugee integration…” there are too many words I don’t understand what you are trying to say. Simplify, for example: “to better understand how refugee integration works” | Thank you for the suggestion. We agree that "due to revolutions" sounds clearer and more appropriate. We’ve made the change accordingly.  Done ✅  Thank you for pointing that out. We have corrected the figure from “26 million refugees” to “42.7 million refugees” and cited the relevant source to ensure accuracy and proper attribution (UNHCR, 2025).  Done ✅  Thank you for your comment.  What we meant is that the quality and nature of refugee education are shaped by the host country's pedagogy and curriculum; for instance, enrollment in Saudi schools exposes refugee students to the Saudi curriculum and educational environment.  Done ✅  Thank you for the comment. Upon review, this remark has been excluded as it was deemed not significant to the content of the study or to improving the quality of the text at this stage  Done ✅  We have reorganized the manuscript into four main sections, given the importance of the information needed to compare the two countries. These sections include: (1) Introduction, (2) scope and study background. We believe this structure enhances the clarity and coherence of the literature review section and better serves the objectives of the study.  Done ✅  Thank you for the comment. We have revised and rewrite the Introduction to be more concise by summarizing the key points instead of detailing every article. We have also updated the in-text citations to follow the correct format as you have suggested.  Done ✅  Thank you for the helpful suggestion. We have simplified this expression to “…to better understand how refugee integration works.”  Done ✅ | Page :3    Page 3  Page 4  Pages : 3 to 12  Page  3-5  Page 10  Lines: 190-191 |
| **15** | You don’t need a “AIMS & RESEARCH” category, you should just write one or two sentences at the end of your introduction to describe the aim of your study.  L164 to 188 not sure you need this paragraph, or it should be reduced and, in the introduction, before the aim. | As suggested, we have deleted the “AIMS & RESEARCH” section.  We have shortened and moved the relevant information that was originally in this section to the scope and study background.  Done ✅ |  |
| **16** | METHODS “this study adopted a descriptive approach as the most appropriate means of elucidating the challenges experienced by refugee..." what are you trying to say? Simplify  L209 “school-aged”? you should give the specific ages of the children you have studied. Is it only primary school or middle school or high school? Or all? What does “consent” mean? “Usage of the data” do you mean “use of the data”?  You should put capital letter at the begging of each sentence L225 “. between” | We have rewritten this sentence regarding your request at the beginning of method section to ensure clarity.  For example: This study adopted a descriptive approach to elucidate the challenges experienced by refugee children aged 4–6 years living in Saudi Arabia and Turkey from their parents’ perspectives*.*  Done ✅  The sentence was rewritten  Done ✅  We have rewritten the whole paragraph.  Done ✅ | Page 12  Page :13 |
| **17** | RESULTS The results section should be factual, with no comparison to other studies or no explanation. For example, L306 to 310 you compare the results to those of another study. This paragraph should be in the discussion L329 to L333 same here, this should be in the discussion section. L353 to 356 same here L369 to 371 same here L375 to 378 same here L404 to 407 same here L461 to 471 same here | Thank you for your detailed feedback. We have reviewed the indicated lines (L306–310, L329–333, L353–356, L369–371, L375–378, L404–407, and L461–471) and moved all comparisons and explanatory content to the discussion section, as recommended. The results section now presents only factual findings.  Done ✅ | Pages 17-26 |
| **18** | Limitations: parents answer for their children, subjective answers so difficult to compare. | Thank you for highlighting this important limitation. We acknowledge that relying on parents’ responses about their children may introduce subjectivity and potentially affect the comparability of the data.We have added a clear discussion of this limitation in the revised manuscript, emphasizing that future research could include direct assessments or observations to complement parental reports and enhance objectivity.  Done ✅ | Page 33 |
| **Reviewer #5** | | | |
| **19** | # 32 Please Use the most recent UNHCR statistics on the refugees and the proportion of refugees that are children. | Thank you for this comment. The statistics have been updated using the most recent data published by the United Nations High Commissioner for Refugees (UNHCR).  Done ✅ | Page 4 |
| **20** | #32-37. this requires further explanation - more is required here introducing the topic of refugee education - among children? youth? | Thank you for your feedback We have revised the Introduction and the background to specify that the study focuses on **refugee education among school-aged children**, highlighting the challenges they face in host countries.  Done ✅ | Pages: 3 to 11 |
| **21** | #34 sentence referring to *refugee communities* should introduce this topic. More is required about how these communities are affected by host societies and then an expansion on refugee children’s education. | Thank you for your comment. We have revised the Introduction to expand on and clarify the text referring to refugee communities at the outset to properly introduce the topic.  Done ✅ | Pages : 3 to 6 |
| **22** | #38 to #48, references are required and a clearer explanation of plural societies. | Thank you for your valuable feedback. We have updated the manuscript to include relevant citations and expanded the explanation to better define the concept of plural societies and their significance in the context of refugee integration.  For example, see reference : **3-16-10**  Done ✅ | Page7 |
| **23** | #49-50 - *In addition to the economic consequences, various issues related to human rights,*  *democracy, citizenship, and education have emerged [3].* what is this sentence introducing?  The introduction requires a greater focus on the challenges of refugees in plural societies and the issue of educating refugee children. | The sentence is introducing challenges faced by host societies. We have changed it to reflect this meaning in a clearer and more accurate way; specifically, we have removed the mention of “economic issues” as this was not an area covered in this study.  Done ✅  The Introduction now includes all the issues of education of refugee children and is combined with the background of the study.  Done ✅ | Page :4  Pages: 3to 11 |
| **24** | Literature review - #74 "*refugee children face several challenges*..."only one challenge was mentioned, please expand on this topic.  #75. Please clarify how Job opportunities and social status is relevant to refugee children or omit this sentence.  Please expand on the enrolment rates of refugee children. | Thank you for your feedback. We have revised this section to elaborate on the multiple challenges refugee children face in the **Scope and study background** subsection.  Done ✅  The sentence has been deleted.  Done ✅  Thank you for the suggestion. We expanded on the enrolment rates of refugee children in both countries included in the study.  Done ✅ | Pages: 3-11  Page 6 |
| **25** | #86 The *Kingdom of Saudi Arabia and Turkey are among the Middle Eastern countries that welcome the most refugees*. in relation to what other nations? Please clarify or change the wording of this sentence.  The % of refugees in Turkey have been quoted twice between #85 and #102 and they are different. Please clarify or connect these two statistics in some way. Are there similar statistics available for Saudi Arabia on school age children. | Thank you for the helpful feedback. We have revised the sentence to clarify the comparison between both countries.  Done✅  Thank you for pointing this out. We have revised the text to clarify the difference between the two statistics. One refers to the overall percentage of refugees residing in both countries, while the other focuses specifically on school-aged refugee children enrolled in the education system.  Done ✅ | Pages: 5-6  Pages :5-6 |
| **26** | You describe Saudi Arabia and Turkey as diverse nations; more information needs to be included on their diversity and refugee / migrant populations and pathways to these countries.  More is required on education policies or policies related to refugee children from Saudi Arabia.  More is required on the nuanced challenges of refugee children and youth of school-age and their educational experiences related to their refugee status. | We have revised the text to include more detailed information on the linguistic, and cultural diversity of both Saudi Arabia  and Turkey.  Done ✅  Thank you for your comment. We have expanded the section to include more detailed information on education-related policies in Saudi Arabia that affect refugee and displaced children, particularly those from Syria  Done ✅  We have expanded the discussion to include a more nuanced analysis of the specific educational challenges faced by refugee children due to their social and cultural conditions the subject of the current study at the Scope and study background section  Done✅ | Pages: 3-11  Pages :5-6  Pages: 6 to 10 |
| **27** | Paragraph #107 to #110 requires referencing | We have added the references.  Reference :12 and 31  Done ✅ | Page 9 |
| **28** | #156 - *Aims and Research Questions* - you really need to be specific about the age range of children you are studying.  When you refer to *'migrant children'* does this include refugees?  #164-165 - requires a reference. More is required about the similarities and differences between Saudi Arabia so that the reader can understand the context of these two countries in relation to the experiences of refugee children and this information should be consolidated and located in one section in the literature review or the introduction. | We have specified the age range of the children in the text.  Age range (4-6)  Done ✅  Yes, when we refer to migrant children, this includes refugees.  Done ✅  We have added credible references to support the claims made regarding refugee enrolment and experiences in both Turkey and Saudi Arabia at the **Scop and study background.**  **Ref: 33- 34 – 35**  Done ✅ | Pages:11 – 12  Pages 9-10 |
| **29** | More is required about the theory developed by Lev Vygotsky, and this should be in the literature review | More details were added  “The current research employs the sociocultural theory perspective, formulated by Vygotsky*”*  Done ✅ | Pages 10-11 |
| **30** | #231 – Can you please clarify in this section and in the aims of the research what the age range of the children that are being referred to.  #237 – More is required when explaining the instrument. A sample question and rating scale is required and how the rating scale is interpreted and what does it mean, especially the second section. All demographic variables that were collected should be listed in the section describing the instrument. | We have specified the age range of the children (4-6).  Done ✅  More details were added to describe the sample selection strategy, 3-point Likert scale, and demographic variables.  Done ✅ | Pages 11 -12    Pages: 12to 16 |
| **31** | #289 – The results section should only state the results of this study in relation to the research questions for the study. Other studies or research reports should be left for the discussion. | We have revised the results section to focus only on this study’s findings. References to other studies were removed and moved to the discussion section  Done ✅ | Pages 17-26 |
| **32** | #321 – when referring to moderate levels of significance – please clarify whether you mean moderate levels of statistical significance or priority in terms of ranking? | The priority or importance ranking implied by the relative weights, not to statistical significance.  Done ✅ |  |
| **33** | #350-#359; #375-378 should be part of the discussion,  please omit from the results section. What should be highlighted here are the similarities and differences between countries in the outcomes of the current study, even though they were not statistically significant, trends should be noted. | We have removed interpretive content from the results section and relocated it to the discussion.  The similarities and differences in the findings of the current study between the two countries were not specifically emphasized or distinguished. Instead, the focus was placed on the cultural, educational, and social challenges faced by refugee children in both countries  Done ✅ | Pages 26-32 |
| **34** | Table 8 and Table 9 and possibly Table 10 should be figures to show patterns of scores related to level of parental education. | We have added figures to show patterns of scores related to level of parental education.  Fig: 4 – 5  Done ✅ | 25-26 |
| **35** | #495 The social and economic characteristics of Turkish and Syrian Families –  If data was collected on this issue this should be highlighted in the results section. It should also be discussed in the introduction if there have been previous studies highlighting this issue. In the discussion more is required about this in the discussion – What did Kirdar et al., and Shuayb et al study exactly? And how does the current study add to that understanding and in relation to refugee children. | Thank you for your insightful comment.  We have added the relevant explanations in the Introduction section and aim of the study.  We have also expanded the Discussion section to include comparisons between our findings and those of Kirdar et al.’s and Shuayb et al.’s studies.  Done ✅ | Page 4  Pages: 29- 31 |
| **36** | #498 Recommendations – more is required here. The outcomes of your study have important implications for policies related to refugee children and teaching practices in schools and with families. Government policies and teaching practices should be discussed separately. | Recommendations related to government policies and teaching practices have been discussed separately.  Done ✅ | Page 31 |
| **37** | You may want to expand your literature review to refugee children of Islamic background in a number of regions and countries that accept immigrants, in order to expand the evidence-base for the consequences of cultural and linguistic differences on the educational experiences of refugee children; and also the shared experience of forced displacement in refugee families that is well documented in research conducted in many other countries. | Thank you for your suggestion. We have expanded the literature review to include studies on refugee children of Islamic background in several countries, highlighting the effects of cultural and linguistic differences and experiences of forced displacement on their educational experiences, including Egypt and Jordan as an examples of Islamic countries  Done ✅ | Page: 7 |
