# Supplementary material for: Beyond borders: Exploring the challenges of refugee children in Saudi Arabia and Turkey
Source: PLoS One. 2025 Nov 19;20(11):e0334841. doi: 10.1371/journal.pone.0334841 (PMC12629453; doi:10.1371/journal.pone.0334841)
Supplement: Peer review history

# Peer review: Decision Letter 0

**DOI:** 10.1371/journal.pone.0334841.r001

**Authors:** Magdalena Szaflarski

19 Jun 2025

PONE-D-24-51686

Beyond borders: Exploring the challenges of refugee children in Saudi Arabia and Turkey

PLOS ONE

Dear Dr. bin Kadasah,

Thank you for submitting your manuscript to PLOS ONE. After careful consideration, we feel that it has merit but does not fully meet PLOS ONE’s publication criteria as it currently stands. Therefore, we invite you to submit a revised version of the manuscript that addresses the points raised during the review process.

This study is timely and extends current literature on refugee children in the Middle East/Southern Europe. There are some gaps in the literature review and discussion, and clarifications are also needed in the methods and results. Additional recommendations address language and editorial improvements. Please respond to comments from Reviewers 2, 4 and 5, and revise the manuscript accordingly.

Please submit your revised manuscript by Aug 02 2025 11:59PM. If you will need more time than this to complete your revisions, please reply to this message or contact the journal office at plosone@plos.org . When you're ready to submit your revision, log on to https://www.editorialmanager.com/pone/ and select the 'Submissions Needing Revision' folder to locate your manuscript file.

Please include the following items when submitting your revised manuscript:

- A rebuttal letter that responds to each point raised by the academic editor and reviewer(s). You should upload this letter as a separate file labeled 'Response to Reviewers'.
- A marked-up copy of your manuscript that highlights changes made to the original version. You should upload this as a separate file labeled 'Revised Manuscript with Track Changes'.
- An unmarked version of your revised paper without tracked changes. You should upload this as a separate file labeled 'Manuscript'.

If you would like to make changes to your financial disclosure, please include your updated statement in your cover letter. Guidelines for resubmitting your figure files are available below the reviewer comments at the end of this letter.

If applicable, we recommend that you deposit your laboratory protocols in protocols.io to enhance the reproducibility of your results. Protocols.io assigns your protocol its own identifier (DOI) so that it can be cited independently in the future. For instructions see: https://journals.plos.org/plosone/s/submission-guidelines#loc-laboratory-protocols . Additionally, PLOS ONE offers an option for publishing peer-reviewed Lab Protocol articles, which describe protocols hosted on protocols.io. Read more information on sharing protocols at https://plos.org/protocols?utm_medium=editorial-email&utm_source=authorletters&utm_campaign=protocols .

We look forward to receiving your revised manuscript.

Kind regards,

Magdalena Szaflarski, PhD

Academic Editor

PLOS ONE

Journal Requirements:

When submitting your revision, we need you to address these additional requirements.

1. Please ensure that your manuscript meets PLOS ONE's style requirements, including those for file naming. The PLOS ONE style templates can be found at

https://journals.plos.org/plosone/s/file?id=wjVg/PLOSOne_formatting_sample_main_body.pdf and

https://journals.plos.org/plosone/s/file?id=ba62/PLOSOne_formatting_sample_title_authors_affiliations.pdf

2. Please provide a complete Data Availability Statement in the submission form, ensuring you include all necessary access information or a reason for why you are unable to make your data freely accessible. If your research concerns only data provided within your submission, please write "All data are in the manuscript and/or supporting information files" as your Data Availability Statement.

3. PLOS requires an ORCID iD for the corresponding author in Editorial Manager on papers submitted after December 6th, 2016. Please ensure that you have an ORCID iD and that it is validated in Editorial Manager. To do this, go to ‘Update my Information’ (in the upper left-hand corner of the main menu), and click on the Fetch/Validate link next to the ORCID field. This will take you to the ORCID site and allow you to create a new iD or authenticate a pre-existing iD in Editorial Manager.

4. Please include captions for your Supporting Information files at the end of your manuscript, and update any in-text citations to match accordingly. Please see our Supporting Information guidelines for more information: http://journals.plos.org/plosone/s/supporting-information.

***Comments from the editorial office:** Upon internal evaluation of the reviews provided, we kindly request you to disregard the reviewer report provided by Reviewer 1 and Reviewer 3. No amendments are required in response to Reviewer 1 and 3’s comments.*

[Note: HTML markup is below. Please do not edit.]

Reviewers' comments:

Reviewer's Responses to Questions

**Comments to the Author**

1. Is the manuscript technically sound, and do the data support the conclusions?

The manuscript must describe a technically sound piece of scientific research with data that supports the conclusions. Experiments must have been conducted rigorously, with appropriate controls, replication, and sample sizes. The conclusions must be drawn appropriately based on the data presented.

Reviewer #1: Yes

Reviewer #2: Partly

Reviewer #3: Yes

Reviewer #4: Partly

Reviewer #5: Yes

**********

2. Has the statistical analysis been performed appropriately and rigorously?

Reviewer #1: Yes

Reviewer #2: Yes

Reviewer #3: Yes

Reviewer #4: I Don't Know

Reviewer #5: Yes

**********

3. Have the authors made all data underlying the findings in their manuscript fully available?

The PLOS Data policy requires authors to make all data underlying the findings described in their manuscript fully available without restriction, with rare exception (please refer to the Data Availability Statement in the manuscript PDF file). The data should be provided as part of the manuscript or its supporting information, or deposited to a public repository. For example, in addition to summary statistics, the data points behind means, medians and variance measures should be available. If there are restrictions on publicly sharing data—e.g. participant privacy or use of data from a third party—those must be specified.

Reviewer #1: Yes

Reviewer #2: Yes

Reviewer #3: Yes

Reviewer #4: Yes

Reviewer #5: Yes

**********

4. Is the manuscript presented in an intelligible fashion and written in standard English?

PLOS ONE does not copyedit accepted manuscripts, so the language in submitted articles must be clear, correct, and unambiguous. Any typographical or grammatical errors should be corrected at revision, so please note any specific errors here.

Reviewer #1: Yes

Reviewer #2: Yes

Reviewer #3: Yes

Reviewer #4: No

Reviewer #5: Yes

**********

5. Review Comments to the Author

Please use the space provided to explain your answers to the questions above. You may also include additional comments for the author, including concerns about dual publication, research ethics, or publication ethics. (Please upload your review as an attachment if it exceeds 20,000 characters)

Reviewer #1: 1.While the literature review is thorough, it could benefit from a more critical analysis of existing studies. For example, the authors could discuss the limitations of previous research and how their study addresses these gaps. The review could also include more recent studies (post-2020) to ensure that the discussion is up-to-date, especially given the rapidly changing nature of refugee crises and policies.

2. The study focuses on Syrian refugee parents, which is understandable given the large Syrian refugee population in both countries. However, the findings could be more generalizable if the sample included refugees from other nationalities, especially in Saudi Arabia, which hosts refugees from various countries (e.g., Yemenis, Rohingyas).

3.The study could also benefit from a more diverse sample in terms of the children's age groups. The current sample focuses on children aged 4–6 years, but including older children (e.g., adolescents) could provide a more comprehensive understanding of the challenges faced by refugee children at different stages of their development.

4. While the study acknowledges the shared Islamic background of the refugees and host countries, it could delve deeper into how cultural and religious similarities and differences impact the integration process. For example, how do cultural practices in Saudi Arabia and Turkey differ, and how do these differences affect refugee children's adaptation?

5.The study relies solely on quantitative data, which limits the depth of understanding of the challenges faced by refugee children. Incorporating qualitative methods, such as interviews with children, parents, and teachers, could provide richer insights into the lived experiences of refugee children and the nuances of their challenges.

6. The study acknowledges some limitations, such as the lack of extensive literature on refugee education in Saudi Arabia and the focus on a specific age group. However, it could also discuss potential biases in the data, such as the reliance on parental perspectives, which may not fully capture the children's experiences.

Reviewer #2: • This study addresses a highly significant issue—the challenges faced by refugee children. Given that refugee children will play a critical role in shaping the future of host societies, it is essential to identify the difficulties they encounter, particularly in the field of education, and to propose appropriate solutions. In this regard, the authors have highlighted an important and timely topic.

• The current graphical abstract is difficult to follow. Choosing a clearer and more structured diagram would improve its accessibility and effectiveness.

• One of the key challenges in this study is the comparison between Saudi Arabia and Türkiye regarding refugee children. As noted in lines 86–88, the vast difference in refugee population sizes complicates the interpretation of results. While Türkiye, Lebanon, and Jordan have relatively comparable refugee numbers, the disparity between Saudi Arabia and Türkiye is significant. This should be emphasized in the study, and careful attention must be paid when interpreting the findings.

• The study relies on outdated sources for both countries, with several references exceeding ten years in age. Such references may not accurately reflect the current socio-political context and evolving educational dynamics. To ensure the validity and relevance of the analysis, it is crucial to incorporate more recent research and updated data.

• In addition, statistical information concerning the countries should be drawn from official government documents or legal reports to ensure factual accuracy and reliability.

• Certain sentences, such as those in lines 173–174, reflect conceptual inaccuracies by conflating religious sects with ethnic groups, which may lead to confusion regarding the social and religious composition of the population.

• The sample selection process should be explained in greater detail, as the current description lacks sufficient clarity. Without this information, there is a risk of potential bias in the study's findings.

• A clearer methodological approach and more detailed explanation are necessary. If the study intended to use a Likert-type questionnaire, this should be explicitly stated. While 'High – Medium – Low' response options may be suitable for assessing intensity or frequency, they are less appropriate for measuring attitudes, beliefs, or satisfaction. In such cases, a standard Likert scale is generally more reliable and widely accepted.

• Since the paper aims to explore educational, social, and cultural challenges, these topics are addressed in a broad manner. To strengthen the study, the scope should be more narrowly defined, and the research approach to each topic should be clearly articulated.

• While the statistical analyses are sufficient to address the stated research questions at a basic level, they are not comprehensive. A more in-depth analysis is needed to enhance understanding and provide deeper insights into the research questions.

• The manuscript brings attention to a highly relevant and significant topic that warrants further academic investigation. The focus of the study is both timely and well-justified. However, to strengthen the overall rigor and clarity of the research, several methodological aspects would benefit from further elaboration. Specifically, greater clarity is needed regarding the criteria and rationale for country selection, the choice of statistical data sources from the literature, the sampling strategy, the explanation of the methodological framework, and the inclusion of more advanced statistical analyses. Addressing these points would enhance the study’s reliability and academic contribution.

Reviewer #3: Peer Review Report for the Manuscript Titled “Beyond Borders: Exploring the Challenges of Refugee Children in Saudi Arabia and Turkey”

Overall Evaluation:

This manuscript presents a valuable and timely investigation into the educational, social, and cultural challenges faced by Syrian Muslim refugee children in Saudi Arabia and Turkey. The comparative dimension offers meaningful academic insight. I commend the author for the effort. However, revisions are needed to improve clarity, methodological robustness, and academic alignment. Detailed comments follow.

1. Title: The current title is stylistically appealing but lacks clarity and specificity. Consider revising it to: Beyond Borders: Educational, Social, and Cultural Challenges of Syrian Muslim Refugee Children in Saudi Arabia and Turkey. This version is more precise and enhances searchability.

2. Abstract: The abstract outlines the study’s aims, methods, and findings but requires two key improvements:

a) Replace generic references to “refugees” with “Syrian Muslim refugee children” to avoid ambiguity.

b) Refine the keywords. Suggested options: Syrian refugee children, educational challenges, social integration, cultural adaptation, Saudi Arabia, Turkey.

3. Introduction: The introduction addresses broad themes such as diversity and education but does not sufficiently narrow the focus early on. The term "refugees" is used generally, which may cause confusion as it implies inclusion of all nationalities, ethnicities, and religious backgrounds. It is important to clarify that the study specifically targets Syrian Muslim refugee children. To improve this section:

a) Clearly define the research target group (Syrian Muslim refugee children) to avoid ambiguity.

b) Include a concise problem statement linking refugee status to educational and cultural integration challenges.

c) Ensure a smooth transition from broad/global issues to the specific national contexts.

d) Clearly state the research problem and objectives.

4. Literature Review: The review provides strong coverage of Turkey but insufficient attention to Saudi Arabia, weakening the comparative dimension. Recommendations:

a) Expand the Saudi context using relevant literature, reports, or policy documents.

b) Develop a comparative framework to highlight key similarities and differences.

c) Consider a thematic structure (e.g., educational policy, integration, language) to improve coherence and analytical depth.

5. Research Objectives and Questions: The objectives are relevant but must consistently refer to “Syrian Muslim refugee children” to ensure clarity. Align the research questions and all empirical sections (methods, results, discussion) with this demographic focus.

6. Methodology and Sample Design: Include a brief narrative description of participant distribution across both countries to complement the table and clarify sample representativeness.

7. Statistical methods: The statistical approach is generally appropriate but needs the following improvements:

a) Clearly state the measurement scale used in the questionnaire (e.g., Likert) and explain how relative weights were calculated.

b) For Q4 (country comparison), the use of an independent samples t-test is appropriate. However, you must test and report its assumptions (normality and homogeneity of variance).

c) For Q5 (differences by parental education), t-tests are not suitable if there are more than two categories. A One-Way ANOVA or a non-parametric equivalent should be used instead.

d) The statistical software used for the analysis should be explicitly mentioned.

e) Report effect size measures to provide a sense of the practical significance of the results, not just statistical significance.

f) Consider a Two-Way ANOVA to explore interaction effects between country of residence and parental education.

6. Descriptive Statistics Results: Current descriptive statistics are not disaggregated by country, limiting comparative insight. Suggestions:

a) Provide a narrative summary of participant distribution across Saudi Arabia and Turkey.

b) Revise Table 1 with a comparative table by host country, including: country of residence, fathers’ and mothers’ education levels, years of residency.

c) Include a narrative interpretation of the comparative data to contextualize key differences.

10. Discussion and Conclusion: The discussion is grounded in relevant literature and highlights major challenges, but its impact can be enhanced:

a) Explicitly relate findings back to the research questions.

b) Emphasize how national contexts shape educational and social experiences.

c) Offer actionable insights for policymakers and educators.

d) Provide a comparative synthesis of key findings.

e) Clearly outline the contribution of the study to the existing literature

f) Propose concrete, data-driven policy recommendations.

11. Study Strengths and Limitations: The exclusive focus on Syrian Muslim refugee children is a strength but also a limitation. Recommendation: Explicitly acknowledge this in the abstract, methodology, and discussion to clarify the scope and generalizability.

Final Recommendation: Accept with Major Revisions

The manuscript addresses a critical and timely topic and has the potential to contribute significantly to the field of refugee education. However, as noted in the above comments, major revisions are required to enhance its conceptual clarity, methodological transparency, and comparative robustness.

I respectfully encourage the author to address the feedback provided and wish them every success in revising and strengthening the manuscript.

Reviewer #4: OVERALL COMMENTS

Interesting subject but I recommend major revisions with a need to focus, shorten and simplify so that the manuscript is easier to read. Please read again carefully to correct all grammar and translations mistakes. I feel like there are many long and complicated sentence, which makes it difficult to read.

Please separate the manuscript into only 4 parts : Introduction, Method, Results, Discussion

SPECIFIC COMMENTS

ABSTRACT

Try to shorten your phrases and be as concise as possible+++

Be more specific about the results. I have trouble understanding this phrase : “The findings revealed various challenges, particularly in education, with differences in severity affected by parental education but not residency.” what severity are you referring too?

You often repeat elements that mean the same thing, seems redundant “universal struggles faced by refugees worldwide” try to simplify : either “universal” or “worldwide”.

I don’t understand the meaning of this phrase “findings suggest the need for support and policy reforms to aid refugee children’s integration into (into what?)”

What do you mean by “governing bodies”?

Some phrases are complicated and difficult to read and understand “this study expands the existing knowledge base…” simplify+++ for exemple : “This study contributes significantly to the existing literature on refugee children and the challenges they face, while providing valuable directions for future research”

INTRODUCTION

“Owing to revolutions….” Is owing the right word? This doesn’t sound correct, you should maybe use the term “due to revolutions” or “as a result of revolutions”

When you cite “26 million refugees” you should give the source

What do you mean by “type and quality of refugee education depends on the context in which they are accomodated”? for example?

“given the dimension… in resolving their education related problems” I feel like this phrase is incomplete, what are you trying to say?

Why is the literature review separated from the introduction? It should be included in in or in the discussion.

Overall, the introduction is too long. You don’t have to explain and detail every articles. You should write the introduction, make a resume and only put their references. If you want to cite an article you should say “Al-Mansour et al. (18)”

Simplify phrases “to gain insights into the nuanced dynamics of refugee integration…” there are too many words i don’t understand what you are trying to say. Simplify, for example : “to better understand how refugee integration works”

You don’t need a “AIMS & RESEARCH” category, you should just write one or two sentences at the end of your introduction to describe the aim of your study.

L164 to 188 not sure you need this paragraph, or it should be reduced and in the introduction, before the aim.

METHODS

“this study adopted a descriptive approach as the most appropriate means of elucidating the challenges experienced by refugee..." what are you trying to say? Simplify

L209 “school-aged”? you should give the specific ages of the children you have studied. Is it only primary school or middle school or high school? Or all?

What does “consint” mean? “Usage of the data” do you mean “use of the data”?

You should put capital letter at the begging of each sentence L225 “. between”

RESULTS

The results section should be factual, with no comparison to other studies or no explanation. For example L306 to 310 you compare the results to those of another study. This paragraph should be in the discussion

L329 to L333 same here, this should be in the discussion section.

L353 to 356 same here

L369 to 371 same here

L375 to 378 same here

L404 to 407 same here

L461 to 471 same here

DISCUSSION

Limitations: parents answer for their children, subjective answers so difficult to compare

Reviewer #5: This is a really important study that addresses the challenges of refugee children in two very common destinations for refugees that are also underrepresented in research related to refugee youth and children.I congratulate the researchers for conducting this study and I have performed and attached a comprehensive review of the article.

**********

6. PLOS authors have the option to publish the peer review history of their article (what does this mean? ). If published, this will include your full peer review and any attached files.

If you choose “no”, your identity will remain anonymous but your review may still be made public.

**Do you want your identity to be public for this peer review?** For information about this choice, including consent withdrawal, please see our Privacy Policy .

Reviewer #1: No

Reviewer #2: No

Reviewer #3: No

Reviewer #4: No

Reviewer #5: No

**********

[NOTE: If reviewer comments were submitted as an attachment file, they will be attached to this email and accessible via the submission site. Please log into your account, locate the manuscript record, and check for the action link "View Attachments". If this link does not appear, there are no attachment files.]

While revising your submission, please upload your figure files to the Preflight Analysis and Conversion Engine (PACE) digital diagnostic tool, https://pacev2.apexcovantage.com/ . PACE helps ensure that figures meet PLOS requirements. To use PACE, you must first register as a user. Registration is free. Then, login and navigate to the UPLOAD tab, where you will find detailed instructions on how to use the tool. If you encounter any issues or have any questions when using PACE, please email PLOS at figures@plos.org . Please note that Supporting Information files do not need this step.

Attachment Submitted filename: PlosOne Article Review.docx

# Author comment: Author response to Decision Letter 1

**DOI:** 10.1371/journal.pone.0334841.r002

8 Sep 2025

Dear Editor,

Thank you for your comment. We have carefully reviewed PLOS ONE’s style guidelines and ensured that our manuscript now complies with all formatting requirements, including proper file naming conventions.

And we have named both the manuscript and figures files, as well as the tables and appendix within the text according to the journal’s naming style. We have also ensured that paper is double-spaced, kept heading levels to a maximum of 3, and revised the references to follows Vancouver style, among others.”

Please note that all requested changes are highlighted in gray in the revised manuscript file.

attached file that contain descriptive responses to the reviewers comments .

Attachment Submitted filename: 20-8 REFUGEE_Response_to_Reviewers__2___1_ (1) (2).docx

# Editor report: Decision Letter 1

**DOI:** 10.1371/journal.pone.0334841.r003

**Authors:** Magdalena Szaflarski

15 Sep 2025

PONE-D-24-51686R1

Beyond borders: Exploring the challenges of refugee children in Saudi Arabia and Turkey

PLOS ONE

Dear Dr. bin Kadasah,

Thank you for submitting your manuscript to PLOS ONE. After careful consideration, we feel that the manuscript has improved significantly, and that the majority of critiques are addressed effectively in this resubmission. A few minor edits are requested:

=======================

1. Lines 185-193: Please clarify that migrant children include refugee children. Also, provide a rationale for the sample ages 4-6 years (lines 192-193).

2. Line 12:  the referent for "their" is unclear. Is it "refugee children's"? Please clarify.

3. Graphical abstract: Please edit the bullet points to all starting with an upper-case letter. Also, last bullet uses a verb structure different from the other bullet points. Please make the form consistent throughout the statements (e.g., no active verb).

=======================

Please submit your revised manuscript by Oct 30 2025 11:59PM. If you will need more time than this to complete your revisions, please reply to this message or contact the journal office at plosone@plos.org . When you're ready to submit your revision, log on to https://www.editorialmanager.com/pone/ and select the 'Submissions Needing Revision' folder to locate your manuscript file.

Please include the following items when submitting your revised manuscript:

- A rebuttal letter that responds to each point raised by the academic editor and reviewer(s). You should upload this letter as a separate file labeled 'Response to Reviewers'.
- A marked-up copy of your manuscript that highlights changes made to the original version. You should upload this as a separate file labeled 'Revised Manuscript with Track Changes'.
- An unmarked version of your revised paper without tracked changes. You should upload this as a separate file labeled 'Manuscript'.

-->If you would like to make changes to your financial disclosure, please include your updated statement in your cover letter. Guidelines for resubmitting your figure files are available below the reviewer comments at the end of this letter.

If applicable, we recommend that you deposit your laboratory protocols in protocols.io to enhance the reproducibility of your results. Protocols.io assigns your protocol its own identifier (DOI) so that it can be cited independently in the future. For instructions see: https://journals.plos.org/plosone/s/submission-guidelines#loc-laboratory-protocols . Additionally, PLOS ONE offers an option for publishing peer-reviewed Lab Protocol articles, which describe protocols hosted on protocols.io. Read more information on sharing protocols at https://plos.org/protocols?utm_medium=editorial-email&utm_source=authorletters&utm_campaign=protocols .

We look forward to receiving your revised manuscript.

Kind regards,

Magdalena Szaflarski, PhD

Academic Editor

PLOS ONE

Journal Requirements:

1. If the reviewer comments include a recommendation to cite specific previously published works, please review and evaluate these publications to determine whether they are relevant and should be cited. There is no requirement to cite these works unless the editor has indicated otherwise. 

2. Please review your reference list to ensure that it is complete and correct. If you have cited papers that have been retracted, please include the rationale for doing so in the manuscript text, or remove these references and replace them with relevant current references. Any changes to the reference list should be mentioned in the rebuttal letter that accompanies your revised manuscript. If you need to cite a retracted article, indicate the article’s retracted status in the References list and also include a citation and full reference for the retraction notice.

[Note: HTML markup is below. Please do not edit.]

[NOTE: If reviewer comments were submitted as an attachment file, they will be attached to this email and accessible via the submission site. Please log into your account, locate the manuscript record, and check for the action link "View Attachments". If this link does not appear, there are no attachment files.]

While revising your submission, please upload your figure files to the Preflight Analysis and Conversion Engine (PACE) digital diagnostic tool, https://pacev2.apexcovantage.com/ . PACE helps ensure that figures meet PLOS requirements. To use PACE, you must first register as a user. Registration is free. Then, login and navigate to the UPLOAD tab, where you will find detailed instructions on how to use the tool. If you encounter any issues or have any questions when using PACE, please email PLOS at figures@plos.org . Please note that Supporting Information files do not need this step.

# Author comment: Author response to Decision Letter 2

**DOI:** 10.1371/journal.pone.0334841.r004

18 Sep 2025

Dear Editor,

We addressed the 3 additional comments from the editor Dr Magdalena Szaflarski, during the manuscript resubmission stage.

few minor edits are requested from the editor

1-Lines 185-193: Please clarify that migrant children include refugee children. Also, provide a rationale for the sample ages 4-6 years (lines 192-193). Thank you for your valuable feedback

We confirm that the term “migrant children” in our manuscript also includes refugee children.

The present study focused specifically on this stage because, Early childhood particularly the period from four to six years of age—represents a critical stage in the development of personal identity. Thus, targeted early interventions during these formative years can play a pivotal role in fostering a strong sense of belonging and facilitating a smoother, more rapid adaptation to newly encountered communities following migration.

2-Line 12: the referent for "their" is unclear. Is it "refugee children's"? Please clarify.

Thank you for pointing out the ambiguity.

We have revised the sentence to clarify the referent. The word “their” specifically refers to refugee children’s adaptation to new cultural and social norms.

3-Graphical abstract: Please edit the bullet points to all starting with an upper-case letter. Also, last bullet uses a verb structure different from the other bullet points. Please make the form consistent throughout the statements (e.g., no active verb). Thank you for the helpful feedback.

We have updated the graphical abstract bullet points so that:

Each bullet point now begins with an upper-case letter.

The last bullet point has been rephrased to match the noun-phrase style of the other bullets (removing the active verb).

4- We have added around [27] recent studies to enhance the content and update the data used, which better represents the current situation in both countries. This has contributed to strengthening the validity and relevance of our analysis. We appreciate your comments, which has helped improve the rigor of the study. We have also removed the cited reference [Pinar W, 2010] because it is outdated and no longer reflects the current state of the literature.

*The recently added studies can be found documented in the reference list at the end of the manuscript

Attachment Submitted filename: REFUGEE_Response_to_Reviewers_.docx

# Editor report: Decision Letter 2

**DOI:** 10.1371/journal.pone.0334841.r005

**Authors:** Magdalena Szaflarski

2 Oct 2025

Beyond borders: Exploring the challenges of refugee children in Saudi Arabia and Turkey

PONE-D-24-51686R2

Dear Dr. bin Kadasah,

We’re pleased to inform you that your manuscript has been judged scientifically suitable for publication and will be formally accepted for publication once it meets all outstanding technical requirements.

Within one week, you’ll receive an e-mail detailing the required amendments. When these have been addressed, you’ll receive a formal acceptance letter and your manuscript will be scheduled for publication.

An invoice will be generated when your article is formally accepted. Please note, if your institution has a publishing partnership with PLOS and your article meets the relevant criteria, all or part of your publication costs will be covered. Please make sure your user information is up-to-date by logging into Editorial Manager at Editorial Manager®  and clicking the ‘Update My Information' link at the top of the page. For questions related to billing, please contact billing support .

If your institution or institutions have a press office, please notify them about your upcoming paper to help maximize its impact. If they’ll be preparing press materials, please inform our press team as soon as possible -- no later than 48 hours after receiving the formal acceptance. Your manuscript will remain under strict press embargo until 2 pm Eastern Time on the date of publication. For more information, please contact onepress@plos.org.

Kind regards,

Magdalena Szaflarski, PhD

Academic Editor

PLOS ONE

Additional Editor Comments (optional):

Reviewers' comments:

# Editor report: Acceptance letter

**DOI:** 10.1371/journal.pone.0334841.r006

**Authors:** Magdalena Szaflarski

PONE-D-24-51686R2

PLOS ONE

Dear Dr. bin Kadasah,

I'm pleased to inform you that your manuscript has been deemed suitable for publication in PLOS ONE. Congratulations! Your manuscript is now being handed over to our production team.

At this stage, our production department will prepare your paper for publication. This includes ensuring the following:

* All references, tables, and figures are properly cited

* All relevant supporting information is included in the manuscript submission,

* There are no issues that prevent the paper from being properly typeset

You will receive further instructions from the production team, including instructions on how to review your proof when it is ready. Please keep in mind that we are working through a large volume of accepted articles, so please give us a few days to review your paper and let you know the next and final steps.

Lastly, if your institution or institutions have a press office, please let them know about your upcoming paper now to help maximize its impact. If they'll be preparing press materials, please inform our press team within the next 48 hours. Your manuscript will remain under strict press embargo until 2 pm Eastern Time on the date of publication. For more information, please contact onepress@plos.org.

You will receive an invoice from PLOS for your publication fee after your manuscript has reached the completed accept phase. If you receive an email requesting payment before acceptance or for any other service, this may be a phishing scheme. Learn how to identify phishing emails and protect your accounts at https://explore.plos.org/phishing.

If we can help with anything else, please email us at customercare@plos.org.

Thank you for submitting your work to PLOS ONE and supporting open access.

Kind regards,

PLOS ONE Editorial Office Staff

on behalf of

Dr. Magdalena Szaflarski

Academic Editor

PLOS ONE
